# SRSF10 Connects DNA Damage to the Alternative Splicing of Transcripts Encoding Apoptosis, Cell-Cycle Control, and DNA Repair Factors

**DOI:** 10.1016/j.celrep.2016.10.071

**Published:** 2016-11-15

**Authors:** Lulzim Shkreta, Johanne Toutant, Mathieu Durand, James L. Manley, Benoit Chabot

**Affiliations:** 1 Département de Microbiologie et d'Infectiologie, Faculté de Médecine et des Sciences de la Santé, Université de Sherbrooke, Sherbrooke, QC J1E 4K8, Canada; 2 Laboratory of Functional Genomics, Faculté de Médecine et des Sciences de la Santé, Université de Sherbrooke, Sherbrooke, QC J1E 4K8, Canada; 3 Department of Biological Sciences, Columbia University, New York, NY, 10027, USA

## Abstract

RNA binding proteins and signaling components control the production of pro-death and pro-survival splice variants of *Bcl-x*. DNA damage promoted by oxaliplatin increases the level of pro-apoptotic Bcl-xS in an ATM/CHK2-dependent manner, but how this shift is enforced is not known. Here, we show that in normally growing cells, when the 5′ splice site of Bcl-xS is largely repressed, SRSF10 partially relieves repression and interacts with repressor hnRNP K and stimulatory hnRNP F/H proteins. Oxaliplatin abrogates the interaction of SRSF10 with hnRNP F/H and decreases the association of SRSF10 and hnRNP K with the *Bcl-x* pre-mRNA. Dephosphorylation of SRSF10 is linked with these changes. A broader analysis reveals that DNA damage co-opts SRSF10 to control splicing decisions in transcripts encoding components involved in DNA repair, cell-cycle control, and apoptosis. DNA damage therefore alters the interactions between splicing regulators to elicit a splicing response that determines cell fate.

## Graphical abstract

Shkreta et al. document a role for SRSF10 in the splicing of the apoptotic regulator *Bcl-x*. Upon DNA damage, the contribution of SRSF10 is enhanced through a change in the interaction of SRSF10 with splicing regulators and the *Bcl-x* pre-mRNA, and is expanded to transcripts encoding DNA damage response components.

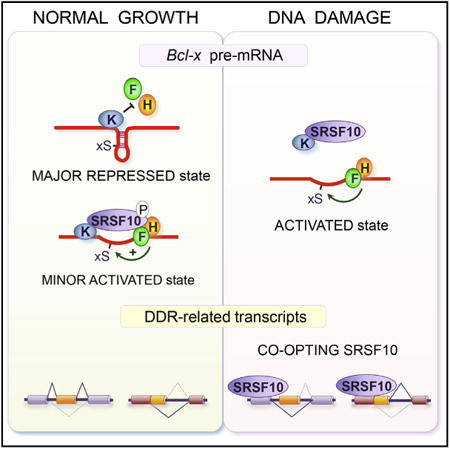

## Introduction

Programmed cell death or apoptosis plays a critical role during animal development and in maintenance of homeostasis (Clavería et al., 2013). Cancer cells often display resistance to signals that elicit apoptosis, yet many anti-cancer strategies aim to generate sufficient DNA damage to override this barrier and ultimately trigger cell death. To design more efficient anti-cancer approaches that will bypass these hurdles, a better understanding of the pathways and molecular mechanisms that lead to apoptosis is required. The function of several apoptotic regulators and effectors is often regulated by alternative splicing to produce variants with activities ranging from pro-apoptotic to pro-survival (Schwerk and Schulze-Osthoff, 2005). At least some of these splicing decisions are coordinated by factors involved in cell-cycle control (Moore et al., 2010). Moreover, DNA damage can reprogram splicing decisions in a variety of cell fate-associated genes including several involved in apoptosis (Dutertre et al., 2014; Naro et al., 2015; Shkreta and Chabot, 2015). For example, DNA damage caused by the topoisomerase inhibitor camptothecin or UV irradiation alters the activity of the Ewing sarcoma protein EWS to affect the alternative splicing of the p53 repressor *MDM2* (Dutertre et al., 2010), the *FAS/CD95* receptor (Paronetto et al., 2014), and genes involved in DNA repair (Paronetto et al., 2011). DNA damage also triggers the formation of a complex between BRCA1 and splicing factors that localizes at DNA repair genes to stimulate co-transcriptional splicing (Savage et al., 2014).

Studies aimed at uncovering regulatory principles of splicing control in apoptotic genes have revealed the contribution of multiple regulators. This is well illustrated with the *Bcl-x* gene (*BCL2L1*), which produces through the use of competing alternative 5′ splice sites (5′ ss), the pro-survival Bcl-xL and the pro-apoptotic Bcl-xS splice variants (Schwerk and Schulze-Osthoff, 2005). More than a dozen splicing factors have been reported to play a role in the control of *Bcl-x* splicing. In normally growing 293 cells, the production of Bcl-xS is strongly repressed by heterogeneous nuclear ribonucleoprotein (hnRNP) K bound immediately upstream of the 5′ss of Bcl-xS (Revil et al., 2009). In contrast, hnRNP F/H proteins act as activators and are recruited immediately downstream of the Bcl-xS 5′ss (Garneau et al., 2005). hnRNP F/H stimulate the 5′ ss of Bcl-xS possibly by preventing the formation of inhibitory G-quadruplexes encompassing the splice site (Dominguez et al., 2010). The binding of RBM25 in exon 2 helps to recruit U1 snRNP to the Bcl-xS 5′ss (Zhou et al., 2008). Both RBM11 and PTBP1 enhance the production of Bcl-xS by preventing the interaction of SRSF1 (Bielli et al., 2014a; Pedrotti et al., 2012). SRSF1 and RBM10, respectively, encourage and repress the production of Bcl-xL (Cloutier et al., 2008; Moore et al., 2010; Paronetto et al., 2007). Core and auxiliary components of the exon-junction complex were identified as repressors of the 5′ss of Bcl-xS (Michelle et al., 2012). Recently, a long non-coding RNA (lncRNA) named INXS was also implicated (DeOcesano-Pereira et al., 2014). INXS is transcribed from the opposite genomic strand of *Bcl-x* and its expression increases the production of Bcl-xS. Upregulation of Sam68 in collaboration with hnRNP A1 promotes Bcl-xS splicing, whereas the Fyn1 tyrosine kinase that targets Sam68 represses it (Paronetto et al., 2007). The transcription factor FBI-1 interacts with Sam68 to reduce its binding to *Bcl-x* transcripts and repress the production of Bcl-xS (Bielli et al., 2014b).

Although a signaling route involving protein kinase C (PKC) enforces the homeostatic repression of Bcl-xS splicing in 293 cells (Revil et al., 2007), more than 20 signaling components affect *Bcl-x* splicing in HeLa cells (Moore et al., 2010). Moreover, the PP1 phosphatase is linked to *Bcl-x* splicing by acting on SF3B1, which represses the production of Bcl-xS (Massiello et al., 2006). Repression of Bcl-xS is lifted following DNA damage. UV irradiation promotes the production of Bcl-xS through an ATM-independent process that changes the speed of elongation of RNA polymerase II (Muñoz et al., 2009). UV exposure also increases INXS expression (DeOcesano-Pereira et al., 2014). The DNA intercalating anti-cancer drugs oxaliplatin and cisplatin switch splicing in favor of Bcl-xS (Shkreta et al., 2008), and this shift occurs through activation of the DNA damage-associated ATM/CHK2 signaling axis (Shkreta et al., 2011).

Here, we document a role for the SR protein SRSF10 in modulating the production of pro-apoptotic Bcl-xS transcripts. In normally growing 293 cells, decreasing and increasing the level of SRSF10 respectively prevent and encourage the production of Bcl-xS. When DNA damage is induced with oxaliplatin, SRSF10 is critical to implement a splicing switch that increases the level of Bcl-xS. Oxaliplatin promotes the dephosphorylation of SRSF10 and prevents SRSF10 and hnRNP K from interacting with the hnRNP F/H-bound *Bcl-x* pre-mRNA. The signaling cascade induced by the DNA damage response therefore converges on SRSF10, likely changing its interaction with hnRNP proteins and the *Bcl-x* pre-mRNA to favor the production of a pro-apoptotic regulator. We show that SRSF10 is required to implement DNA damage-induced splicing shifts in other transcripts encoding components involved in apoptosis, cell-cycle control, and DNA repair, indicating that SRSF10 connects DNA damage with the alternative splicing of transcripts that determine cell fate.

## Results

### SRSF10 Controls Bcl-x Splicing

**Figure 1:**
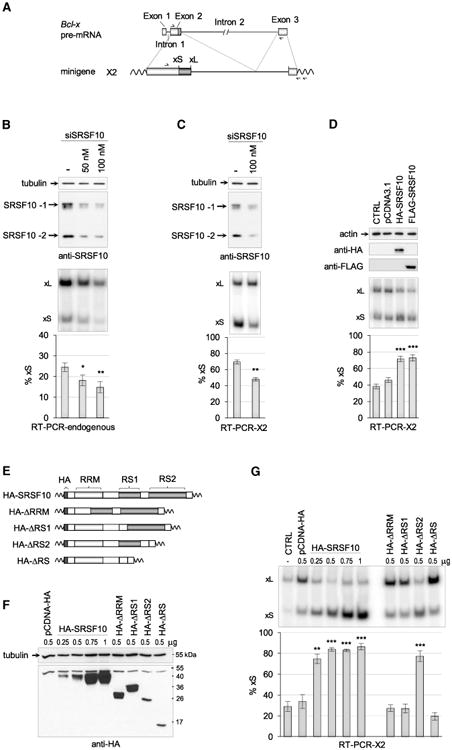
SRSF10 Controls the Alternative Splicing of Bcl-x Schematic representation of the human *Bcl-x* gene (*BCL2L1*) with relevant portions included in minigene X2, and the positions of the RT primer and PCR primer pairs used to carry RT-PCR assays. (B) Following RNAi in 293 cells with the indicated concentrations of siSRSF10, an immunoblot with anti-SRSF10 antibodies was carried out (top panel). The positions of the full–length (SRSF10-1) and truncated splice variant (SRSF10-2) are shown. RT-PCR assays performed on the endogenous *Bcl-x* transcripts; the separation of radio-labeled RT-PCR products is shown for one experiment in the middle panel, with the positions of the Bcl-xS and Bcl-xL products indicated. The histograms shown in the bottom panel represent the average production of Bcl-xS in percentage from triplicates with SDs. (C) Assay as in (B), except that 293 cells were transfected with the *Bcl-x* minigene X2, and the RT-PCR assay used a minigene-specific pair of primers. (D) Plasmids allowing expression of HA-SRSF10 and FLAG-SRSF10 were co-transfected with minigene X2. The impact on *Bcl-x* splicing was monitored as in (C). The CTRL sample was only transfected with X2, whereas pCDNA3.1 is an empty expression plasmid co-transfected with X2. (E) Schematic representation of the HA-SRSF10 and derivatives lacking various domains. (F) After transfection in 293 cells (quantity of plasmid transfected indicated in micrograms), the expression of HA-SRSF10 and derivatives was verified by immunoblotting using anti-HA antibody. (G) Samples of (F) were tested for *Bcl*-x splicing using the X2 reporter as described in (D). Error bars indicate SD. In all cases, asterisks represent significant p values (two-tailed Student's t test)comparing the means between samples and their respective controls; *p < 0.05, **p < 0.01, and ***p < 0.001.

*Bcl-x* is alternatively spliced to produce two variants: the short pro-apoptotic Bcl-xS and the longer anti-apoptotic Bcl-xL (Figure 1A). As part of a screen to identify RNA binding proteins that control *Bcl-x* splicing, we noted that the small interfering RNA (siRNA)-mediated depletion of SRSF10 in 293 cells decreased the relative level of transcripts encoding the pro-apoptotic Bcl-xS variant. Although the impact of depleting SRSF10 is statistically significant, the amplitude of the change was relatively small (approximately 10 percentage points at the highest concentration of siRNA) (Figure 1B). A similar decrease was observed when the depletion of SRSF10 was tested on transcripts expressed from the *Bcl-x* minigene X2 (Figure 1C). To test the effect of increasing the level of SRSF10, we ectopically expressed a HA-tagged and a FLAG-tagged SRSF10 in 293 cells; both versions stimulated the relative level of Bcl-xS transcripts derived from the X2 minigene by nearly 30 percentage points (Figure 1D).

SRSF10 contains one N-terminal RNA-recognition domain (RRM) necessary and sufficient for sequence-specific RNA binding and two C-terminal arginine- and serine-rich domains (RS1 and RS2) involved in protein-protein interactions (Shin et al., 2005). To investigate which domains are required for the activity of SRSF10 on *Bcl-x* splicing, we produced a set of HA-SRSF10 variants lacking one or several domains (Figure 1E). Expression of the variants was verified by immunoblotting with an anti-HA antibody (Figure 1F). The activity of SRSF10 on *Bcl-x* splicing was completely lost when the RRM or the RS1 domain was deleted (Figure 1G). In contrast, deletion of the C-terminal end of SRSF10 that contains the RS2 domain did not prevent activity. Thus, the N-terminal portion of SRSF10 that contains the RRM1 and the RS1 domains is sufficient for modulating *Bcl-x* splicing.

### SRSF10 Control of Bcl-x Splicing Requires hnRNP F/H

**Figure 2:**
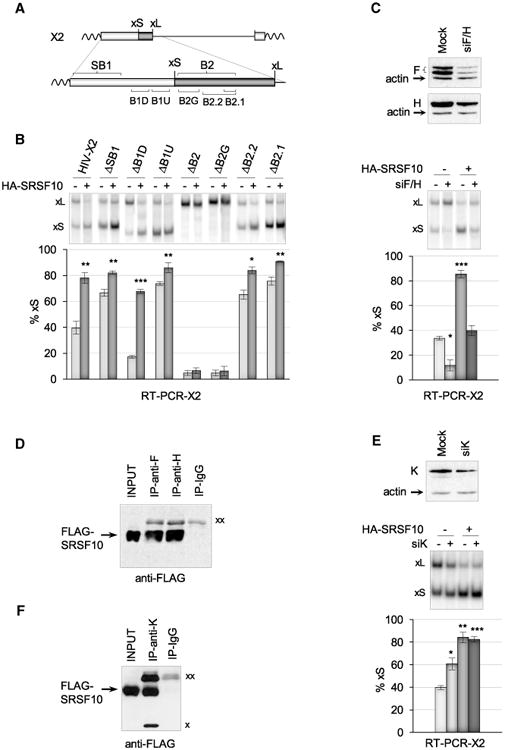
SRSF10 Requires hnRNP F/H to Control Bcl-x Splicing and Interacts with hnRNP F, H, and K Proteins (A) Schematic representation of regulatory elements surrounding the 5′ splice site of Bcl-xS. (B) The X2 minigene and derivatives were transfected either alone or with the HA-SRSF10 plasmid. RT-PCR assays were carried out, and radiolabeled RT-PCR products were fractionated on a non-denaturing gel to quantitate Bcl-xS and Bcl-xL products. The histograms represent the average percentage of Bcl-xS products from triplicate experiments. (C) siF/H was used to deplete hnRNP F/H with an immunoblot shown to verify depletion. HA-SRSF10 was transfected in 293 cells treated or not with siF/H. RT-PCR was carried out to detect the *Bcl-x* splice variants, as described in (B). (D) Immunoprecipitation assays using 293 cells transfected with FLAG-SRSF10. The material recovered was fractionated and transferred on nitrocellulose decorated with anti-FLAG antibodies. (E) siK was used to deplete hnRNP K and an immunoblot is shown. HA-SRSF10 was transfected in 293 cells treated or not with siK. RT-PCR was carried out to detect the *Bcl-x* splice products, as described in (*B*). (F) Immunoprecipitation assays with hnRNP K antibody. Using 293 cells transfected with FLAG-SRSF10, the recovered material was fractionated, transferred on nitrocellulose that was decorated with anti-FLAG antibodies. “xx” and “x” indicate the large and small immunoglobulin subunits that react with the secondary antibody, respectively. Error bars indicate SD. Asterisks represent p values (two-tailed Student's t test) comparing the means between samples and their respective controls; *p < 0.05, **p < 0.01, and ***p < 0.001.

To assess whether SRSF10 acts through a defined sequence element, we tested a set of *Bcl-x* minigenes carrying individual deletions of previously identified regulatory elements flanking the competing 5′ splice sites (Figure 2A). As shown in Figure 2B, the deletion of each element had the expected impact on *Bcl-x* splicing. For all deletions, except that of B2 and B2G, HA-SRSF10 stimulated the level of Bcl-xS to or near the maximal amount produced from the wild-type *Bcl-x* construct, indicating that B2G is the minimal element required for the SRSF10-induced splicing shift. B2G is bound by the hnRNP F and H proteins to enhance Bcl-xS splicing (Garneau et al., 2005). Notably, the SRSF10-induced production of Bcl-xS was compromised by the siRNA-mediated depletion of hnRNP F/H (Figure 2C; from an average of 52 percentage points in the controls to an average of 28 percentage points in the siF/H-treated samples). The statistical significance of this difference (two-tailed t test with p value of 0.012) indicates that hnRNP F/H proteins are important for modulation of *Bcl-x* splicing by SRSF10.

As the RRM domain of SRSF10 is essential for activity (Figure 1G), SRSF10 may bind to the *Bcl-x* pre-mRNA. Consistent with this view, antibodies against SRSF10 recovered the *Bcl-x* pre-mRNA from a cell extract (see below). However, a gel-shift assay did not detect a stable interaction between recombinant SRSF10 and a 223-nt-long *Bcl-x* RNA that includes B2G (Figure S1A). Although GA-rich motifs that represent binding sites for SRSF10 are absent in B2G, putative high-affinity binding sites in the SB1 element (Figure S1B) are not required for the SRSF10-induced splicing shift (Figure 2B). Thus, SRSF10 may interact with other portions of the *Bcl-x* pre-mRNA, or its association with the pre-mRNA may occur or be stabilized by interaction with other RNA binding proteins.

Because the impact of SRSF10 on *Bcl-x* splicing requires hnRNP F/H, SRSF10 may interact with hnRNP F/H. To test this hypothesis, we performed an immunoprecipitation assay using extracts from 293 cells expressing FLAG-SRSF10. Extracts were pre-treated with ribonuclease A to eliminate interactions that occur through RNA bridging. The immunoblot reveals that anti-hnRNP F and anti-hnRNP H antibodies recovered FLAG-SRSF10 (Figure 2D), indicating that SRSF10 is physically associated with hnRNP F/H (estimated at 0.5%–1% of the total amount of SRSF10, based on input level and recovery by immunoprecipitation). The reciprocal immunoprecipitation performed with anti-FLAG recovered hnRNP F (Figure S2A). hnRNP F and H also interact with endogenous SRSF10 (Figures S2B–S2D).

hnRNP K interacts with element B1U immediately upstream of the 5′ ss of Bcl-xS to repress it in 293 cells (Figure 2A) (Revil et al., 2009). The depletion of hnRNP K using RNAi increased the production of Bcl-xS made from X2 (Figure 2E) and the endogenous *Bcl-x* transcripts (Figure S2F) (Revil et al., 2009). As a consequence, the amplitude of the response to HA-SRSF10 was reduced from 44 to 22 percentage points when hnRNP K was partially depleted (p value of 0.04 using a two-tailed t test) (Figure 2E). These results and the observation that HA-SRSF10 further stimulates the production of Bcl-xS when hnRNP K is partially depleted may be explained if SRSF10 helps relieve repression by hnRNP K, and that it neutralizes the K proteins that remain after partial depletion. Likewise, deleting B1U activates the 5′ss of Bcl-xS, but HA-SRSF10 offered weak but significant stimulation (Figure 2B), possibly because it antagonizes the impact of a hnRNP K binding site in the B1D region (Revil et al., 2009). A physical interaction between SRSF10 and hnRNP K is supported by the observation that an immunoprecipitation assay using anti-hnRNP K antibodies and an RNase-treated extract recovered FLAG-SRSF10 (Figure 2F). Based on input and recovery levels, 0.7% of FLAG-SRSF10 is estimated to be in interaction with hnRNP K. This interaction with hnRNP K also occurs with endogenous SRSF10 (Figures S2C, S2D, and S2E). Thus, over-expression of HA-SRSF10 relieves the repression conferred by hnRNP K, and this effect may occur through a direct interaction of SRSF10 with hnRNP K and hnRNP F/H.

### DNA Damage Alters the Interaction of SRSF10 with Splicing Regulators and the *Bcl-x* Pre-mRNA

**Figure 3:**
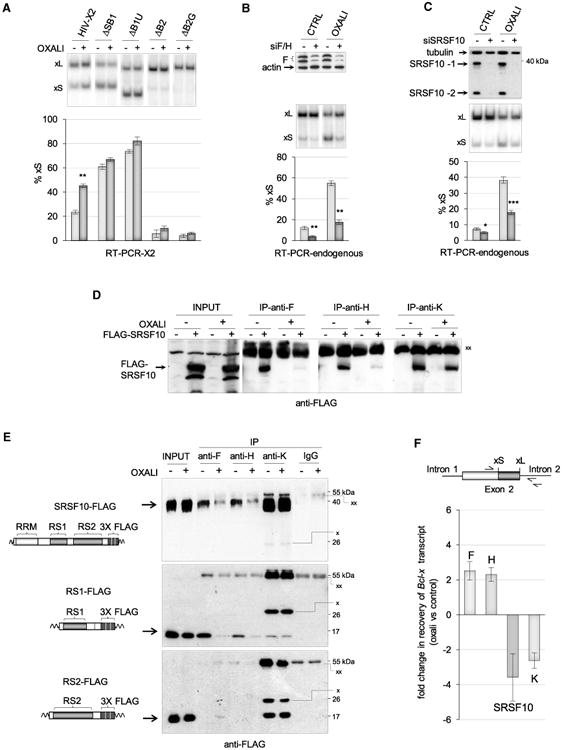
The Bcl-x Splicing Shift Induced by Oxaliplatin Requires SRSF10 and hnRNP F/H, and Is Associated with the Loss of Interaction between SRSF10 and hnRNP F/H (A) The X2 minigene and derivatives lacking different elements were transfected into 293 cells. Four hours later, cells were treated with oxaliplatin for 24 hr. The impact of oxaliplatin on *Bcl-x* splicing was determined by RT-PCR. (B) The role of hnRNP F/H was tested by depleting hnRNP F/H by RNAi. The top panel shows an immunoblot for hnRNP F. The middle and bottom panels show the results of the RT-PCR assays to detect endogenous *Bcl-x* transcripts. (C) The role of SRSF10 on the oxaliplatin-induced *Bcl-x* splicing shift was tested by depleting siSRSF10. The top panel confirms the depletion of SRSF10. The middle and bottom panels show the results of the RT-PCR assays on endogenous *Bcl-x* transcripts. (D) Immunoprecipitation of FLAG-SRSF10 was performed with anti-hnRNP F, H, and K antibodies using extracts prepared from cells treated or not with oxaliplatin, and expressing or not FLAG-SRSF10. The input content of FLAG-SRSF10 is shown and represents 1/50^th^ of the samples used for immunoprecipitation. Immunoprecipitates were fractionated on gel and proteins were transferred to nitrocellulose decorated with anti-FLAG antibodies. “xx” indicates the large immunoglobulin subunit used for the immunoprecipitation that reacts with the secondary antibody. (E) The immunoprecipitation assays used cells expressing SRSF10-FLAG, RS1-FLAG, or the RS2-FLAG treated or not with oxaliplatin. The procedure is as described in (D). Anti-K antibodies are from mouse, whereas anti-F and anti-H antibodies are from rabbit. A rabbit IgG was used for the control immunoprecipitation. “xx” and “x” indicate the large and small immunoglobulin subunits that react with the secondary anti-mouse antibody, respectively. (F) Immunoprecipitation was carried out on cells treated or not with oxaliplatin. The recovered RNA was quantitated for *Bcl-x* pre-mRNA using primers shown on the top. Raw data are provided in Table S1. The differential between values obtained for each antibody comparing the impact of oxaliplatin is plotted in histograms. The result obtained with IgG control immunoprecipitations is provided in Table S1. Also shown in Table S1 are the results obtained from two experiments comparing immunoprecipitations performed with cells treated with formaldehyde and untreated cells.

Repression in the production of pro-apoptotic Bcl-xS is lifted when a genotoxic stress is applied to 293 cells. For example, oxaliplatin shifts splicing to Bcl-xS by activating the DNA damage response (DDR) pathway (Shkreta et al., 2011). The 361-nt regulatory region SB1, located 150 nt upstream of the Bcl-xS 5′ss (Figure 2A), like the B1U element bound by hnRNP K, is required for repression of the 5′ss of Bcl-xS (Revil et al., 2007; Shkreta et al., 2011); when either the B1U element or the SB1 region is removed, oxaliplatin fails to further stimulate Bcl-xS splicing (Figure 3A). To achieve its function, SB1 may communicate with regulators bound close to the Bcl-xS 5′ss. Consistent with this view, the B2G element, which is required for the activity of hnRNP F/H and SRSF10, is essential for the oxaliplatin-mediated splicing switch (Figure 3A). Likewise, the oxaliplatin-induced splicing switch is compromised when the level of either hnRNP F/H or SRSF10 is reduced by RNAi (Figures 3B and 3C). In the case of hnRNP F/H, the oxaliplatin shift decreases 3-fold from an average of 43 to an average of 13 percentage points (p value < 0.0001 by two-tailed t test), whereas in the case of SRSF10, the oxaliplatin shift decreases 2.5-fold from an average of 31 to an average of 13 percentage points (p value < 0.0001 by two-tailed t test). Thus, hnRNP F/H and SRSF10 contribute to enforce the use of the 5′ ss of Bcl-xS when the DDR pathway is activated by oxaliplatin.

Given that SRSF10 interacts with hnRNP F/H and hnRNP K, we asked whether oxaliplatin affects these interactions. First, we observed that oxaliplatin does not change the expression level of SRSF10, hnRNP F, and hnRNP K (Figures S3A and S3B). Likewise, the depletion of SRSF10 did not affect the expression of hnRNP F and K, nor did the depletion of hnRNP F/H or K greatly affect the expression of SRSF10 (Figures S3C and S3D). Second, we performed immunoprecipitation assays with anti-F, anti-H, and anti-K antibodies. The results indicate that the interaction between SRSF10 and hnRNP K is maintained when cells are treated with oxaliplatin (Figure 3D). In contrast, the interaction between SRSF10 and hnRNP F and H was nearly completely lost in oxaliplatin-treated cells (Figure 3D). To identify RS domains of SRSF10 that contribute to the interaction with hnRNP F/H, and whose ability to interact may be altered by oxaliplatin, we used FLAG-RS1 and RS2 derivatives (Figure 3E). Notably, the RS1 but not the RS2 domain of SRSF10 interacts with hnRNP F/H, and the interaction of RS1 with both hnRNP F and hnRNP H was sensitive to oxaliplatin (Figure 3E). In contrast, hnRNP K interacts with both RS domains, and these interactions are not disrupted by oxaliplatin (Figure 3E). These results suggest that the RS1 domain contains residues that contribute to the dynamic interaction of SRSF10 with hnRNP F/H.

To address whether oxaliplatin also affects the interaction of each of the above factors with the *Bcl-x* pre-mRNA, we used qRT-PCR to measure the amount of *Bcl-x* RNA recovered by immunoprecipitation with antibodies against hnRNP F, H, K, and FLAG. The reverse transcriptase primer and one PCR primer were designed to map in the intron downstream of the Bcl-xL 5′ss to ensure that interactions with the pre-mRNA rather than the mRNA were monitored (Figure 3F; Table S1). The recovered material was treated with DNase I to eliminate a potential contribution of contaminating genomic DNA. As shown in Figure 3F and Table S1, oxaliplatin decreased the association of SRSF10 and hnRNP K with the *Bcl-x* pre-mRNA, but increased the interaction of hnRNP F and H.

**Figure 4:**
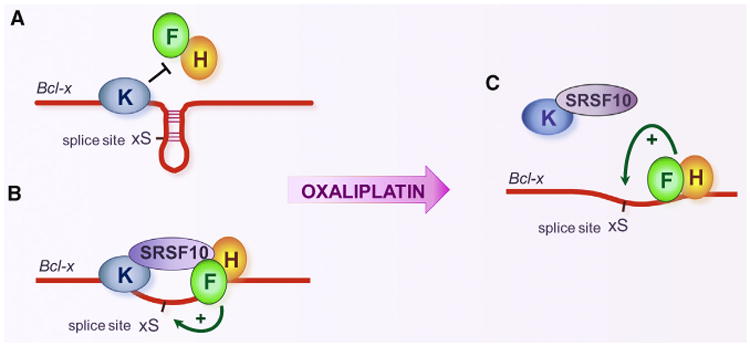
Schematic Model of the Proposed Role of SRSF10, hnRNP F/H, and hnRNP K in *Bcl-x* Pre-mRNA Splicing, and How Oxali-platin Reprograms Their Interactions Repressor complex representing the major regulatory assembly in normally growing 293 cells. (B) Activating complex proposed to form on a minor fraction of *Bcl-x* transcripts in normally growing 293 cells or when SRSF10 is overex-pressed. (C) Impact of oxaliplatin on the interaction of regulatory components leading to the activation of the 5′ss of Bcl-xS.

We propose the following model to explain the results of the overexpression, depletion, and immunoprecipitation assays. In normal growth conditions, hnRNP K represses the 5′ss of Bcl-xS on a majority of transcripts (Figure 4A). The mechanism of this repression is not known, but hnRNP K may antagonize the binding of hnRNP F/H that is required to make the 5′ss of Bcl-xS structurally available (Dominguez et al., 2010; Garneau et al., 2005). By associating with hnRNP K on a small fraction of transcripts, SRSF10 may neutralize repression by encouraging the recruitment of hnRNP F/H to promote 5′ss recognition (Figure 4B). In normally growing 293 cells, the impact of depleting SRSF10 or hnRNP F/H is small because the 5′ss of Bcl-xS on most transcripts is repressed by hnRNP K (Figure 4A). Oxaliplatin reduces the binding of hnRNP K and SRSF10 to the *Bcl-x* pre-mRNA, and although the association of SRSF10 with hnRNP K is maintained, the interaction of SRSF10 with hnRNP F/H is disrupted. This reconfiguration may be important to keep hnRNP K from being recruited to the pre-mRNA and would explain why the production of Bcl-xS is compromised when SRSF10 is depleted in oxaliplatin-treated cells. The reduced recruitment of hnRNP K would facilitate hnRNP F/H binding and stimulate splicing to the 5′ss of Bcl-xS (Figure 4C).

### Oxaliplatin Affects the Phosphorylation of SRSF10 and hnRNP K

**Figure 5:**
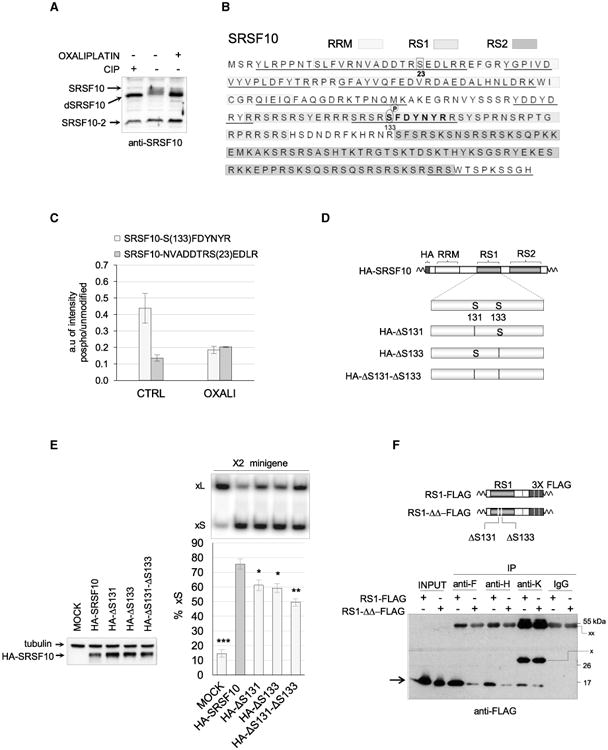
Dephosphorylation of SRSF10 by Oxaliplatin (A) Total cellular extracts were collected 24 hr after treatment or not with oxaliplatin. Aliquots of the untreated cellular extract were incubated with or without calf intestinal phosphatase (CIP) for 15 min at 37°C. Proteins were fractionated on gel and transferred to nitrocellulose to reveal SRSF10. (B) Amino acid sequence of the SRSF10-1 protein showing the different domains (RRM, RS1, and RS2) in differently shaded boxes. The regions of SRSF10 to which peptides identified by LC-MS/MS analysis mapped are underlined.(C) Proteins recovered from duplicate anti-FLAG immunoprecipitations using cells expressing FLAG-SRSF10 and treated or not with oxaliplatin were analyzed by LC-MS/MS analysis after trypsin digestion. Histograms depict the relative abundance of two SRSF10 peptides containing a phosphorylated serine compared to the respective unmodified versions. The peptide in the RS1 domain is shown in bold in (B), and the position of both serines is indicated. (D) Diagram of the mutated versions of HA-SRSF10 carrying either a deletion of S131, S133, or both. (E) The impact of mutated HA-SRSF10 on *Bcl-x* splicing was tested by co-transfecting minigene X2 and carrying out RT-PCR assays. The percentage of Bcl-xS is represented in histograms with asterisks indicating p values when samples are compared to wild-type HA-SRSF10. Error bars indicate SD. *p < 0.05, **p < 0.01, and ***p < 0.001. (F) The anti-F, anti-H, and anti-K immunoprecipitations used extracts from cells expressing RS1-FLAG or RS1-ΔΔ-FLAG (structure diagrammed on top). The procedure is as described in Figure 3E. A rabbit IgG was used in the control immunoprecipitation.

Given that oxaliplatin activates DDR signaling (Shkreta et al., 2011), and that phosphorylation controls the activity of SRSF10 (Shin et al., 2004, 2005; Shin and Manley, 2002), we hypothesized that oxaliplatin may affect the phosphorylation of SRSF10. The immunoblot performed to evaluate the expression of SRSF10 following oxaliplatin treatment showed the presence of faster migrating forms that are consistent with dephosphorylation (Figure S3B). We repeated this gel fractionation after treating a cell extract with calf intestinal phosphatase (CIP), which converts endogenous SRSF10 into faster gel-migrating forms (Figure 5A), matching previous observations (Shi and Manley, 2007). Treating 293 cells with oxaliplatin also converted endogenous SRSF10 into faster migrating forms (Figure 5A). To identify peptides in SRSF10 that become dephosphorylated when cells are treated with oxaliplatin, we performed an anti-FLAG immunoprecipitation in duplicate using cells expressing FLAG-SRSF10, and subjected the recovered material to liquid chromatography-tandem mass spectrometry (LC-MS/MS) analysis after trypsin digestion. SRSF10 peptides covered approximately 40% of the protein (Figure 5B). Of these, peptide SFDYNYR carries a serine at position 133 (RS1 domain) that was phosphorylated in 40% of all the SFDYNYR peptide recovered in untreated samples (in bold in Figure 5B). The relative recovery of the phosphorylated version of this peptide was reduced 2-fold in samples derived from cells treated with oxaliplatin (Figure 5C). In contrast, the relative level of a different peptide carrying a phosphorylated serine mapping in the RRM domain of SRSF10 was not significantly affected by oxaliplatin (Figure 5C). Thus, oxaliplatin promotes the dephosphorylation of FLAG-SRSF10 at serine 133.

Based on the gel migration profile of SRSF10 from oxaliplatin-treated cells (Figure 5A), other dephosphorylation events likely contribute to the strong effect of oxaliplatin on the migration of SRSF10. Given that our peptide coverage underrepresents the RS1 and RS2 domains (Figure 5B), our assessment of the phosphorylation status of SRSF10 is likely to be incomplete. Nevertheless, we assessed the functional contribution of serine 133 by producing a deletion variant (HA-ΔS133; Figure 5D). Given the presence of another serine at position 131, we also mutated this position individually and in combination with serine 133 (HA-ΔS131 and HA-ΔS131-ΔS133; Figure 5D). When cotransfected in 293 cells with the *Bcl-x* minigene, HA-ΔS131 and HA-ΔS133 displayed a reduced capacity at stimulating the production of Bcl-xS, whereas the double deletion had a stronger effect (Figure 5E). HA-ΔS131-ΔS133 had a similar impact on endogenous *Bcl-x* transcripts (Figure S4). Changing S131 and S133 to alanines also compromised activity but not replacing them with the phosphomimetic aspartate (Figure S4). However, having aspartate at these two positions did not compromise the impact of oxaliplatin (data not shown), possibly indicating that additional dephosphorylated residues make important contributions. Because oxaliplatin affects the phosphorylation of SRSF10 in a domain (RS1) that is important for its activity and its interaction with other splicing regulators, we tested the impact of deleting both S131 and S133 on the ability of RS1 to interact with hnRNP proteins. Strikingly, the mutations strongly compromised the ability of RS1 to interact with hnRNP F and hnRNP H, and slightly reduced the interaction with hnRNP K (Figure 5F).

hnRNP K displays a reduced ability to bind to the *Bcl-x* pre-mRNA when cells are treated with oxaliplatin (Figure 3F). Because the interaction of hnRNP K with phosphatase 2A inhibitor protein SET increases the nucleic acid binding of hnRNP K (Almeida et al., 2014), dephosphorylation of hnRNP K may reduce its binding to the *Bcl-x* pre-mRNA. To determine whether oxaliplatin affects the phosphorylation of hnRNP K, we recovered endogenous hnRNP K in cells treated or not with oxaliplatin, and subjected the recovered material to LC-MS/MS analysis after trypsin digestion. We identified one phosphorylated peptide specific to hnRNP K, and its recovery relative to the non-phosphorylated version was reduced 2-fold in cells treated with oxaliplatin (Figure S5). This peptide contains a phosphoserine at position 216 that maps in between the KH2 and RGG domains. Because phosphorylation of serine 216 contributes to the transcriptional activity of hnRNP K, its dephosphorylation may also decrease binding to the *Bcl-x* pre-mRNA.

### SRSF10 Connects DNA Damage with the Alternative Splicing of Transcripts Implicated in the DDR

**Figure 6:**
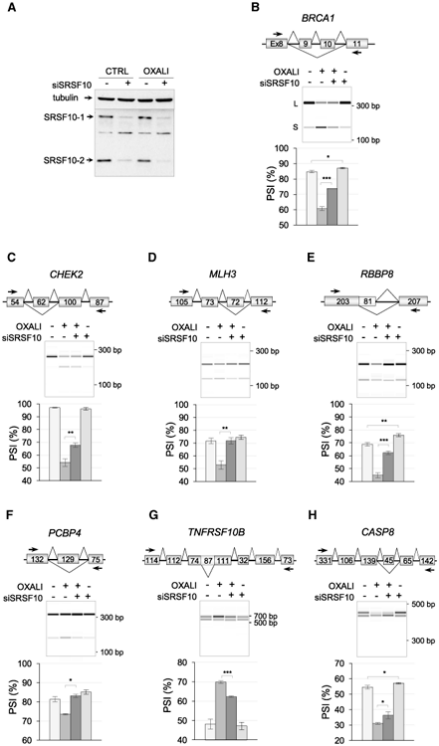
Impact of the Depletion of SRSF10 on the Oxaliplatin-Induced Splicing Shifts (A) Immunoblot showing the siRNA-mediated depletion of SRSF10 in cells treated or not with oxaliplatin. (B–H) For each subsequent panel, the name of the gene and the structure of its relevant portion are shown. (B) *BRCA1*. Exon numbers are shown. (C) *CHEK2*. Size of exons in nucleotides. (D) *MLH3*. Size of exons in nucleotides. (E) *RBBP8*. Size of exons in nucleotides. (F) *PCBP4*. Size of exons in nucleotides. (G) *TNFRSF10B*. Size of exons in nucleotides. (H) *CASP8*. Size of exons in nucleotides. In each panel, the RT-PCR analysis presents electrophero-grams with molecular weight markers. Triplicate experiments are shown as histograms with percent splicing index (PSI). Error bars indicate SD. Asterisks indicate significant *P value*s obtained when comparing control with siSRSF10 or samples treated with oxaliplatin with samples treated with both siSRSF10 and oxaliplatin; *p < 0.05, **p < 0.01, and ***p < 0.001.

The activity of splicing regulatory factors is often altered by DNA damage possibly to coordinate the splicing regulation of genes involved in cell-cycle control, DNA repair, and apoptosis (Shkreta and Chabot, 2015). To determine whether SRSF10 regulates splicing of other transcripts encoding proteins implicated in the DDR, we tested genes involved in apoptosis, cell-cycle control, and DNA repair, and identified 28 events whose alternative splicing was sensitive to oxaliplatin (Δpercent splicing index [ΔPSI] ≥ 5 percentage points with p values < 0.05; Table S2; CTRL–OXALI column). Of these, 13 had their oxaliplatin-mediated shift partially abrogated by the depletion of SRSF10 (Table S2; OXALI–OXALI^si^ column). In addition to *Bcl-x*, seven units had significantly smaller amplitude in the oxaliplatin-induced shift when SRSF10 was depleted (Figure 6). For example, oxaliplatin reduced the skipping of exons 9–10 in *BRCA1* by 24 percentage points but only by 13 percentage points when SRSF10 was depleted (Figure 6B; p value of 0.0027 using two-tailed t test). Statistically significant differences were also obtained for units in *CHEK2*, *MLH3*, *RBBP8*, *PCBP4*, *TNFRSF10B*, and *CASP8* (Figure 6; Table S2). In contrast, of the 43 units that did not respond to oxaliplatin, only *BCLAF1* and *AKIP1* were regulated by SRSF10 (Table S3), suggesting that SRSF10 preferentially controls units that respond to DNA damage. Interestingly and in contrast to *Bcl-x*, the association of FLAG-SRSF10 with the *BCLAF1* and *AKIP1* pre-mRNAs was not affected by oxaliplatin (Table S4), indicating that the oxaliplatin-mediated drop in the association of SRSF10 with the *Bcl-x* pre-mRNA did not occur on non-oxaliplatin-responsive transcripts.

Notably, for 10 of the 13 units sensitive to oxaliplatin that react to a depletion of SRSF10, the impact of this depletion was more important in oxaliplatin-treated cells than in control cells (i.e., ΔPSI between 6 and 17 percentage points in [OXALI–OXALI^si^] relative to ΔPSI of 2 to 7 percentage points in [CTRL–CTRL^si^] (Table S2; Figure 6). Thus, for seven alternative splicing units and *Bcl-x*, the regulatory impact of SRSF10 becomes more important when cells are treated with oxaliplatin.

Several units sensitive to both oxaliplatin and the depletion of SRSF10 reside in genes encoding components involved in apoptosis, DNA repair, and cell-cycle control, and hence are associated with the DNA damage response. Oxaliplatin stimulated the production of a *BRCA1* variant lacking exons 9 and 10 (Figure 6B) that encode a linker region separating the RING domain from the multiple protein interaction platform. *RBBP8* encodes an endonuclease that controls cell-cycle G_2_/M checkpoints and that interacts with BRCA1 to regulate the activation of CHK1. It is not known whether the splice variants of *RBBP8* display different activities. The intron retention event in *TNFRSF10B* promoted by oxaliplatin adds a 29-amino acid segment whose functional impact is not known, as is the case for the CASP8 variants. The checkpoint kinase CHK2 is normally activated upon DNA damage to induce cell-cycle arrest (Matsuoka et al., 1998), and oxaliplatin promotes the inclusion of an exon in *CHEK2* that would produce a truncated version through frameshift. The fact that SRSF10 is required for the DNA-damage-induced shifts in *CHEK2* suggests that SRSF10 may help override cell-cycle checkpoints. In combination with the increased production of pro-apoptotic Bcl-xS, the net effect may accelerate commitment toward apoptosis. Although the precise function of many of the above splice variants in our cell system remains to be assessed, a recent analysis in chicken DT-40 cells suggested a role for SRSF10 in controlling the splicing of transcripts of genes that belong to these functional categories; alternative splicing of *Bap1* (BRCA1-associated protein), *Cdk13* and *Casp1* were in the top 12 events controlled by SRSF10 (Zhou et al., 2014b). Likewise, transcripts that code for proteins linked to apoptosis (e.g., *BCLAF1* and *RAC1*) form a top functional category controlled by SRSF10 in human RKO cells (Zhou et al., 2014a). Inspecting the sequence of SRSF10-regulated exons and their flanking introns for the presence of GA-motifs did not reveal an over-representation of putative SRSF10 binding sites relative to randomly selected alternative splicing units that do not respond to a depletion of SRSF10 (Figure S6). To determine whether the SRSF10-dependent response to oxaliplatin may implicate regulators that interact with SRSF10 to control *Bcl-x* splicing, we tested whether hnRNP F/H and hnRNP K were also contributing to regulation. Notably, all units, except *RBBP8*, were regulated by hnRNP F/H, and two units (*RBBP8*, *PCBP4*) were controlled by hnRNP K (Figure S7). Depleting hnRNP F/H altered the response to oxaliplatin for three units (*BRCA1*, *CHEK2*, and *TNFRSF10B*). This result suggests that coordination of oxaliplatin-induced splicing shifts often implicate the combinatorial contribution of SRSF10, hnRNP F/H, and hnRNP K.

## Discussion

We have documented a role for SRSF10 in *Bcl-x* splicing. In normally growing 293 cells, only small amounts of the pro-apoptotic Bcl-xS splice variant are made. The overexpression of SRSF10 encourages the production of Bcl-xS, but this effect is prevented when hnRNP F/H are depleted or when the sequence to which they bind, immediately downstream of the 5′ ss of Bcl-xS, is removed. Because SRSF10 interacts with hnRNP F/H and the repressor protein hnRNP K, our results suggest that SRSF10, hnRNP F/H, and hnRNP K are part of a complex that attenuates repression of the 5′ss of Bcl-xS (Figure 4B). hnRNP K-mediated repression likely occurs on the bulk of *Bcl-x* pre-mRNAs (Figure 4A), whereas the SRSF10-mediated anti-repression may be effective only on a small fraction of *Bcl-x* transcripts.

Treating 293 cells with oxaliplatin elicits a large increase in the production of pro-apoptotic Bcl-xS, and both SRSF10 and hnRNP F/H are required for this splicing shift to occur. Oxaliplatin abrogates the interaction of SRSF10 with hnRNP F/H, and leaves the SRSF10/hnRNP K interaction unaffected. Moreover, oxaliplatin decreases the association of both SRSF10 and hnRNP K with the *Bcl-x* pre-mRNA, but increases that of hnRNP F/H. These results suggest that oxaliplatin prevents the association of a SRSF10/hnRNP K complex with the *Bcl-x* pre-mRNA, allowing hnRNP F/H to bind to *Bcl-x* transcripts and enforce the production of Bcl-xS. Because hnRNP F helps maintain the G-rich environment of the Bcl-xS 5′ ss in a single-stranded conformation (Dominguez et al., 2010), hnRNP F/H may facilitate U1 snRNP binding to this splice site. Consistent with the view that the DDR activates a cascade of signaling events often converging on splicing regulators (Dutertre et al., 2014; Shkreta and Chabot, 2015), we identified a phosphoserine in hnRNP K that becomes dephosphorylated when cells are treated with oxaliplatin. Although the impact of this modification remains to be evaluated, the interaction of hnRNP K with the phosphatase inhibitor protein SET increases its binding to ssDNA (Almeida et al., 2014), in line with the notion that the dephosphorylation of hnRNP K may reduce RNA binding. Our mass spectrometry analysis also identified a phosphoserine (Ser133) in SRSF10 that becomes dephosphorylated when cells are treated with oxaliplatin. Ser133 is located in the RS1 domain of SRSF10. RS1 is essential for the activity of SRSF10, and by itself can interact with hnRNP F/H and hnRNP K, and mimic the oxaliplatin-mediated changes in these interactions sustained by SRSF10. Deleting Ser133 and the nearby Ser131, or substituting them for alanines, reduced the ability of SRSF10 to control *Bcl-x* splicing. Moreover, the deletion of both Ser131 and Ser133 strongly decreased the ability of the RS1 domain to interact with hnRNP F/H. The above dephosphorylation events associated with the activity of oxaliplatin fit well with our previous observation that phosphatases contribute to the action of oxaliplatin on *Bcl-x* splicing (Shkreta et al., 2011). Although we have not demonstrated that the dephosphorylation of SRSF10 directly contributes to the *Bcl-x* splicing shift, overall, our results are consistent with a model whereby DNA damage triggers the dephosphorylation of SRSF10 and hnRNP K to reduce their interaction with the *Bcl-x* pre-mRNA and the hnRNP F/H proteins, allowing the latter to stimulate the 5′ss of Bcl-xS (Figure 4).

Under normal growth conditions, multiple signaling routes converge on *Bcl-x* splicing (Moore et al., 2010; Revil et al., 2007). Ser133 in the RS1 domain of SRSF10 is part of an environment that matches the consensus phosphorylation sites of several kinases, including PKC, which is implicated in the homeostatic regulation of *Bcl-x* splicing in 293 cells (Revil et al., 2007). Upon DNA damage, these signaling routes may be altered and new ones may become activated. Previously, we showed that oxaliplatin affects *Bcl-x* splicing through ATM/CHK2 signaling and the activation of phosphatases (Shkreta et al., 2011). The oxaliplatin-mediated inhibition of kinases that phosphorylate SRSF10, such as SRPK1 and SRPK2, is unlikely because cisplatin activates SRPK1/2 (Edmond et al., 2011). Oxaliplatin may promote the dissociation of 14-3-3 proteins, which protect SRSF10 from dephosphorylation (Shi and Manley, 2007). However, the RS2 domain of SRSF10, which contains residues important for the interaction with 14-3-3 proteins (Shi and Manley, 2007), is not required for modulation of *Bcl-x* splicing. Thus, the precise signaling route affected by oxaliplatin that leads to altered SRSF10 and hnRNP K function remains to be identified. Moreover, it is unclear whether this signaling route is operative in other cell lines. Although oxaliplatin shifts *Bcl-x* splicing in all cancer cell lines tested so far (Shkreta et al., 2008), as PKC signaling does not contribute to *Bcl-x* splicing control in the cancer cell lines that we have tested (Revil et al., 2007), it will be worth exploring whether the signaling network that controls SRSF10 phosphorylation also operates in cancer cell lines.

We cannot rule out that oxaliplatin affects the activity of other factors controlling *Bcl-x* splicing. SRSF2 stimulates the production of Bcl-xS (Merdzhanova et al., 2008) in H358 and A459 cells, and cisplatin increases the activity of SRSF2 (Edmond et al., 2011). In HeLa and 293 cells, however, the RNAi-mediated knockdown of SRSF2 does not significantly affect *Bcl-x* splicing (Papasaikas et al., 2015) (data not shown). Because SRSF1 stimulates the 5′ss of Bcl-xL (Cloutier et al., 2008; Paronetto et al., 2007), its repression would increase Bcl-xS. However, UV and cisplatin increase the activity of SRSF1 in MCF-7 and HeLa cells, respectively (Comiskey et al., 2015). Whereas Sam68 collaborates with hnRNP A1 to favor the production of Bcl-xS in HEK293 cells (Paronetto et al., 2007), the topoisomerase inhibitor methoxantone and UV provoke the accumulation of Sam68 in nuclear granules and the retention of hnRNP A1 in the cytoplasm, respectively (Busà et al., 2010; van der Houven van Oordt et al., 2000). If oxaliplatin similarly changes the localization of Sam68 and hnRNP A1, Bcl-xS production should decrease, in contrast to what we observed. Finally, although UV slows RNA polymerase II elongation to promote the production of Bcl-xS, this pathway is independent of ATM/ATR and is not used when cells are treated with doxorubicin (Muñoz et al., 2009). The impact of oxaliplatin on transcription elongation remains to be evaluated.

Our results therefore provide a detailed description of how the DDR interfaces with regulatory factors to control alternative splicing decisions on a gene that determines cell fate. The modulation of protein-protein and protein-RNA interactions by DNA damage has so far been documented only for the splicing regulator EWS; UV promotes a relocalization of EWS associated with a reduction in its interaction with target transcripts, whereas camptothecin and cisplatin disrupt the interaction of EWS with YB-1 to affect transcription-coupled *Mdm2* splicing (Dutertre et al., 2010; Paronetto et al., 2011). The recent demonstration of the existence of large splicing regulatory complexes containing RBFOX proteins and other regulatory hnRNP proteins such as hnRNP H and M proteins (Damianov et al., 2016) is consistent with the multiple interactions between splicing regulators that were uncovered in our study. Whether the composition of these complexes is systematically reconfigured by various stresses is an intriguing question that remains to be assessed.

### SRSF10 Modulates the Splicing Response to DNA Damage

The DDR activates a signaling network that coordinates DNA repair with the cell cycle, and with apoptosis when damage is too extensive. Although many elements of this response operate rapidly by post-translationally modifying components of these machineries, a slower route implements regulatory changes in transcription and translation. DDR-mediated changes in splice site selection is increasingly recognized as another important path that controls the activity of machineries that sense, repair, and react to DNA damage (Dutertre et al., 2014; Naro et al., 2015; Shkreta and Chabot, 2015). Genotoxic agents or treatments have a broad impact on the splicing and alternative splicing of transcripts encoding proteins involved in DNA repair, cell-cycle control, and apoptosis (reviewed in the study by Shkreta and Chabot, 2015). However, the splicing regulatory mechanisms affected by the DDR are less well understood. UV, cisplatin, and the topoisomerase II inhibitor etoposide increase the expression or phosphorylation of SR proteins and modulate the alternative splicing of target transcripts (Comiskey et al., 2015; Edmond et al., 2011; Leva et al., 2012). UV also alters the level of phosphorylation of RNA polymerase II to affect the speed of transcription and splice site selection (Muñoz et al., 2009). In one recent example, etoposide was shown to promote the phosphorylation of chromatin-bound BRCA1 to recruit spliceosomal proteins and stimulate splicing of transcripts from the DNA repair genes *ATRIP*, *BACH*, and *EXO1* (Savage et al., 2014). In many cases, genotoxic stresses change the localization of splicing regulatory factors (Shkreta and Chabot, 2015). For example, DNA damage partially relocalizes EWS to the nucleoli (Paronetto et al., 2011), affecting alternative splicing in the same direction as a depletion of EWS (Dutertre et al., 2010; Paronetto et al., 2011). This situation may also be true for RBMX, FUS, SKIP, and Tra2, whose individual depletions, like that of EWS, increase DNA damage-induced apoptosis (Adamson et al., 2012; Best et al., 2014; Chen et al., 2011; Dutertre et al., 2010; Li et al., 2007; Paronetto et al., 2011).

Here, we have uncovered a mechanism by which DNA damage controls alternative splicing of transcripts encoding proteins involved in apoptosis, cell-cycle control, and DNA repair. Although depletion of SRSF10 compromised several oxaliplatin-induced splicing shifts, depleting SRSF10 by itself only had a modest or no impact on the splicing of these transcripts, suggesting that SRSF10 is co-opted by the DDR to control a broad set of splicing decisions. Based on our analysis of the role of SRSF10 in *Bcl-x* splicing, its transformation into a more efficient splicing regulator is associated with dephosphorylation, a process that maintains its interaction with hnRNP K but decreases its interaction with hnRNP F/H and with the *Bcl-x* pre-mRNA. This regulatory strategy may similarly be applied to the control of other SRSF10-dependent splicing units that respond to oxaliplatin because hnRNP K and hnRNP F/H were implicated in the splicing control of three and eight alternative splicing units (out of nine tested), respectively. Although SRSF10 was originally described as a general splicing repressor activated by dephosphorylation, phosphorylated SRSF10 can also function as a splicing activator (Feng et al., 2008; Shin and Manley, 2002). Our results suggest that the modulating properties of SRSF10 may vary according to the splicing events that are interrogated. Consistent with this view, SRSF10 controls the alternative splicing of exon 5a in *BCLAF1* in a variety of cancer cell lines (Zhou et al., 2014a). The fact that this *BCLAF1* splicing event is not affected by oxaliplatin (Figure S8) suggests that SRSF10 operates through different molecular mechanisms. Thus, SRSF10 controls a complex functional network because it suppresses splicing during heat shock and M phase (Shin et al., 2004; Shin and Manley, 2002), controls alternative splicing decisions that elicit myoblast differentiation and glucose production, alters the oncogenic properties of cancer cells (Wei et al., 2015; Zhou et al., 2014a), and modulates the production of splice variants implicated in apoptosis, cell-cycle control, and DNA repair as part of the cellular response to DNA damage.

## Experimental Procedures

### Plasmid Construction, Transfection

Plasmids expressing *Bcl-x* reporter mini-genes and 3XFLAG-SRSF10, FLAG-SRSF10, and HA-SRSF10 were previously described (Cloutier et al., 2008; Garneau et al., 2005; Revil et al., 2009; Shi and Manley, 2007; Shkreta et al., 2011). Plasmids expressing SRSF10 and mutants were produced by PCR site-directed mutagenesis. pcDNA3.1-HA-SRSF10 or p3XFLAG-V14-SRSF10 were used as PCR templates for Pfu-Turbo polymerase and respective primers listed in Table S5. Products were cleaved with BamHI and EcoRI and inserted into pCDNA3.1-HA, or with BglII and EcoRV and inserted into p3XFLAG-V14. Transfections were carried out with polyethyleneimide (Polysciences) or Lipofectamine 2000 (Invitrogen).

### RNAi Assays

Knockdown of *SRSF10* was performed with the siGENOME SMARTpool-Human SRSF10 (2914-02-0005; Dharmacon). The siRNAs against hnRNP F/H (GAACUGAACAAUUUCUUCC) and hnRNP K (UGAUACUCAAUAUGCGCUC) were from previously published work (Garneau et al., 2005; Revil et al., 2009) and synthesized by IDT. siRNAs were transfected (100 nM) using Lipofectamine 2000 (Invitrogen). Proteins or RNA were extracted 72 hr post-transfection.

### Cell Culture and Drugs

Human 293 cells (EcR-293; Invitrogen) were grown at 37°C (5% CO_2_)in DMEM supplemented with 10% fetal bovine serum (FBS). Oxaliplatin was obtained from the Centre de Chimiothérapie-CHUS.

### Immunoblot Analysis

Whole-cell extracts were prepared by lysing cells in Laemmli sample buffer. Equal amounts of total protein were fractionated on SDS-PAGE, and standard protocols were applied for western blotting. Proteins were revealed with primary antibodies against HA-tag (Roche; 12CA5), FLAG (Sigma; F3165), SRSF10 (Abcam; ab77209), hnRNP F or hnRNP H (kindly provided by Doug Black), hnRNP K (kindly provided by G. Dreyfuss), actin (Sigma; A5316), tubulin (ab4074; Abcam), using peroxidase-conjugated secondary antibodies and ECL detection reagent (Amersham). Secondary antibodies were either polyclonal anti-rabbit (Cell Signaling; 7074) or anti-mouse (Bio-Can; 115-035-003).

### RNA Extraction and RT-PCR Analysis

Total RNA was extracted from treated or transfected cells with TRIzol (Invitrogen) as described by the manufacturer. The splicing profile of endogenous or mini-gene-derived *Bcl-x* pre-mRNA was assessed by RT-PCR (Shkreta et al., 2011). The RT-PCR analysis of other genes was performed by the RNomics platform (Sherbrooke). Primers are listed in Table S6.

### RNA Immunoprecipitation and qRT-PCR Analysis

EcR-293 cells treated with 20 μM oxaliplatin for 24 hr. After washing with PBS, the cell pellet was resuspended into RIPA buffer supplemented with protease and RNase inhibitors. Cells were lysed by sonication and the insoluble material was removed by centrifugation at 4°C. The supernatant was precleared by incubation for 1 hr at 4°C with Protein G Sepharose 4 Fast Flow beads (GE Healthcare) previously blocked with yeast tRNA. An aliquot of the precleared supernatant was used as input while the remaining material was used for immunoprecipitation. Precleared whole-cell lysates of equal protein quantities were incubated overnight at 4°C with protein G Sepharose beads coated with antibodies against hnRNP F, H, K, and FLAG. Beads were collected by centrifugation at 1,300 × *g* for 1 min, washed four times with RIPA buffer, resuspended in elution buffer (1% SDS, 5 mM EDTA, 10 mM DTT, 50 mM Tris-HCl, pH 7.4). RNA was extracted using TRIzol, resuspended in 15 μL of H_2_O, treated with DNase I for 15 min at 37°C, and quantitated by spectrometry. Equal quantities of RNA were reverse transcribed using M-MuLV enzyme and the primer X-Int2-1-REV (CAG AGG CCA AAG AAA AGG GAC ACA) annealing in intron 2 of *Bcl-x*. qPCR was carried out using SYBR green (2× Power SYBR Green master mix; ABI; 4367660) and primers X-Int2-2-REV (CAC ACA AGG GGC TTG GTT CTT A) and X-EX-S1-FWD (TCA CCC CAG GGA CAG CAT ATC). The method used to determine the relative abundance of *Bcl-x* pre-mRNA in immunoprecipitates compared Ct using the input sample (pre-immunoprecipitated) as reference, while the difference between control and oxaliplatin-treated samples was calculated using the 2^−ΔΔ^*^C^*^t^ method and was expressed as fold change of *Bcl-x* pre-mRNA recovered from oxaliplatin-treated samples versus the non-treated control.

### Protein Immunoprecipitation and Mass Spectrometry Analysis

EcR-293 cells expressing or not FLAG-SRSF10 and treated or not with oxaliplatin were cultured in 150-mm plates. Collected cells were washed two times with ice-cold PBS and lysed on ice for 30 min in NET-2 buffer (50 mM Tris-HCl, pH 7.4, 150 mM NaCl, 0.05% [vol/vol] Nonidet P-40 added with EDTA-free protease and phosphatase inhibitors cocktail [Roche Diagnostics]). The clarified lysates were supplemented with RNase A solution (0.1 mg/ml of cellular lysate) and incubated at room temperature for 30 min. Aliquots of SureBeads protein G magnetic beads (Bio-Rad) were coupled with antibodies against hnRNP-F, H, K, or monoclonal anti-FLAG M2 antibody (Sigma; F3165) through rotation for 1 hr at room temperature. Equal aliquots of antibody-coupled beads were added to equal amounts of protein containing pre-cleared cell lysates. After overnight incubation at 4°C, beads were magnetized and washed four times with NET2 buffer. Beads were resuspended in Laemmli buffer before gel fractionation. For mass spectrometry analyses, beads were washed four times with 20 mM NH_4_HCO_3_, resuspended in 50 μL of 20 mM NH_4_HCO_3_ buffer containing 1 μg of Trypsin Gold (Promega), and incubated overnight at 37°C while shaking. The reaction was stopped by adding formic acid (1% final). The supernatant was transferred to a new tube, while beads were resuspended in 50 μL of a solution containing 60% acetonitrile, 0.1% formic acid, and incubated for 5 min at room temperature. Both supernatants were pooled and lyophilized. Peptides were resuspended in 30 μL of 0.1 % of trifluoroacetic acid and desalted using Zip Tip C18 (Millipore). Eluted peptides were lyophilized and resuspended in 25 mL of 1% formic acid. Trypsin-digested peptides loaded onto an Acclaim PepMap100 C18 column (Dionex Corporation) were separated using a Dionex Ultimate 3000 nanoHPLC system. The HPLC system was coupled to an OrbiTrap QExactive mass spectrometer (Thermo Fisher Scientific) via an EasySpray source. Data acquired using the Xcalibur software were processed using the MaxQuant software package, version 1.4.1.2, as described previously (Cox and Mann, 2008) employing the Human Uniprot database.

## Supplementary Material

supp_guide

## Highlights

- SRSF10 is involved in the production of Bcl-xS in normal cells and following DNA damage
- DNA damage reconfigures how SRSF10 interacts with hnRNP proteins and the *Bcl-x* pre-mRNA
- A change in the phosphorylation of SRSF10 is associated with this reconfiguration
- DNA damage co-opts SRSF10 to control splicing decisions in other DDR-related transcripts
