# Supplementary material for: SRSF10 Connects DNA Damage to the Alternative Splicing of Transcripts Encoding Apoptosis, Cell-Cycle Control, and DNA Repair Factors
Source: Cell Rep. Author manuscript; Available in PMC 2017 Jun 26. (PMC5483951; doi:10.1016/j.celrep.2016.10.071)

Cell Reports, Volume *17*

Supplemental Information

SRSF10 Connects DNA Damage to the Alternative Splicing of Transcripts Encoding Apoptosis, Cell-Cycle Control, and DNA Repair Factors

Lulzim Shkreta, Johanne Toutant, Mathieu Durand, James L. Manley, and Benoit Chabot

Supplemental legends

Figure S1 (related to Figure 2). SRSF10 binding to *Bcl-x* sequences. (*A*) A gel-shift assay was performed to monitor the interaction of recombinant His-tagged SRSF10 and hnRNP F with ^{32}P-labeled 223 nt RNA containing B2G (map shown on *top*). (*B*) Sequence of *Bcl-x* gene from the beginning of exon 2 to 100 nt downstream of the 5'ss of Bcl-xL. The position of the 5'ss of Bcl-xS is indicated as well as the SB1 and B2G elements. The GA-motifs that represent putative high-affinity (red) and medium-affinity (green) binding sites for SRSF10 were queried using the RBPmap (Paz et al., 2014) and the RBPDB (Cook et al., 2011) programs.

Figure S2 (related to Figure 2). *(A)* Confirming the SRSF10/hnRNP F interaction. An immunoprecipitation assay was carried out using 293 cells transfected with FLAG-SRSF10. The material recovered with the anti-FLAG antibody was fractionated on a denaturing gel and transferred on nitrocellulose that was decorated with the anti-hnRNP F antibody. "xx" indicates the large immunoglobulin subunit that react with the secondary antibody. *(B-E)* Immunoprecipitation were carried out using extracts of 293 cells and anti-F, anti-H and anti-K antibodies. The blots were decorated with an antibody against SRSF10 to reveal the presence of endogenous SRSF10. Based on input used, the fraction of endogenous SRSF10 interacting with hnRNP F/H and hnRNP K is estimated at 0.25-0.40%, and 0.55% respectively. (*F*) Knockdown of hnRNP K affects endogenous *Bcl-x* splicing in 293 cells. 50 nM of siRNA against hnRNP K was used and a RT-PCR of endogenous Bcl-x splice variants was carried out.

Figure S3 (related to Figure 3). Expression of hnRNP F, K and SRSF10. 293 cells were treated with 20 μM of oxaliplatin. After 24 hours, control (non-treated) and oxaliplatin-treated cells were collected and divided into two aliquots. (*A*) One aliquot was used for RNA extraction and radioactive RT-PCR determination of the relative abundance of the Bcl-xS splice variant (*B*) The second aliquot was lysed and the same quantities of extracts were fractionated on a SDS-PAGE gel and transferred onto nitrocellulose membrane. Western blot was performed to decorate the membrane with antibodies against hnRNP K, hnRNP F, SRSF10 and GAPDH. (*C*) Following the depletion of SRSF10 (confirmed in the left portion), the blot was decorated with anti-F and anti-K antibodies (right portion). (*D*) Following the depletion of hnRNP F/H and hnRNP K (confirmed in the top immunoblots), the blot was decorated with the anti-SRSF10 antibody.

Figure S4 (related to Figure 5). Impact of SRSF10 mutants on *Bcl-x* splicing. (*A*) Diagram of the mutated versions of HA-SRSF10 carrying conversion of S131 and S133 to alanine (A) or aspartate (D), or their deletion. (*B*) A western blot showing the expression of tubulin and HA-tagged proteins is shown. (*C*) The impact of expressing mutated HA-SRSF10 on endogenous *Bcl-x* splicing was tested by RT-PCR assays. The percentage of Bcl-xS is represented in histograms with asterisks indicating *P* values when samples are compared to wild-type HA-SRSF10. * = $P < 0.05$.

Figure S5 (related to Figure 5). Dephosphorylation of hnRNP K by oxaliplatin. Proteins recovered from anti-hnRNP K immunoprecipitation was performed in duplicates using 293 cells expressing FLAG-SRSF10 and treated or not with oxaliplatin. The recovered material was analyzed by LC-MS/MS analysis after trypsin digestion. Histograms represent the relative abundance of a hnRNP K peptide containing a phosphorylated serine compared to its respective unmodified version in each set of samples.

Figure S6 (related to Figure 6). Frequency of the putative SRSF10 binding sites in alternative splicing units. The first group includes ten alternative splicing units that react to oxaliplatin in a SRSF10-dependent manner (*Bcl-x* and splicing units shown in Fig. 6). The second group includes 10 splicing units that did not respond to oxaliplatin and/or SRSF10 (ΔPSI smaller than 10%) (panel *A*). (*B*) Using RBPmap (Paz et al., 2014) and RBPDB (Cook et al. 2011), each splicing unit (alternative exon and 180 nt of the upstream and downstream introns) was analyzed for putative SRSF10 binding motifs that score as low or threshold of 0.5, medium or threshold of 0.7 and high or threshold of 0.85. The motifs recovered by RBPmap and RBPDB for each category were pooled and the total numbers of motifs are normalized per 100 nt of analyzed sequence. Mean values and their respective standard deviations are plotted for each group.

Figure S7 (related to Figure 6). Impact of knocking down of hnRNP F/H and hnRNP K on the alternative splicing of units that respond to oxaliplatin in a SRSF10-dependent manner.

(*A*) Immunoblots showing the siRNA-mediated depletion of hnRNP F, hnRNP H and hnRNP K in cells that were treated or not with oxaliplatin. For each successive panel (*B-H*), the results of triplicate experiments are shown as histograms with percent splicing index (PSI) indicating the percentage of the larger of the two products over the total. Asterisks indicate *P* values that were significant; * = $P < 0.05$, ** = $P < 0.01$ and *** = $P < 0.001$. In *BRCA1, CHEK2* and TNFRSF10B, the amplitude of the shifts elicited by oxaliplatin were significantly affected when hnRNP F/H proteins were depleted.

Figure S8 (related to Figure 6). Impact of oxaliplatin and the depletion of SRSF10 on the alternative splicing of *BCLAF1* exon 5a. The upper panel is a schematic presentation of exon 5a alternative splicing unit in *BCLAF1*. The positions of primers used to carry the RT-PCR assays are indicated. The results of triplicate assays are shown in lower panel as histograms with percent splicing index (PSI) indicating the percentage of the larger of the two products over the two products.

Table S1 (related to Figure 3F). Compilation of RT-qPCR values obtained for immunoprecipitation of *Bcl-x* pre-mRNA with antibodies targeting various RNA binding proteins. The anti-Flag antibody was used to recover FLAG-SRSF10. RTqPCR was performed on the same amount of RNA for input and immunoprecipitated material from both conditions (oxaliplatin-treated and untreated). Ct values for all tests are indicated. After calculating the ΔCt for each pair of IP/input, ΔΔCt values were calculated for oxaliplatin-treated/untreated pair, and their averages were used to obtain a fold-difference value, as described in Experimental Procedures. IP-Assay #1 and IP-Assay #2 were performed with cells treated with formaldehyde (CROSSLINKED samples) or not treated (NO-CROSSLINK samples). In these two assays a control antibody (IgG) was also used. IP-Assay #2 was performed with a different reverse primer for qPCR (1-REV) also mapping in the *Bcl-x* intron. Values obtained with IP-Assays #3 to #8 were used to produce Figure 3F.

**IP-Assay # 1**	**Ct SYBR**	**CROSSLINKED** samples. cDNA-Primer 3-REV, PCR-Primers 3-REV + 8-FWD											
		1-CTRL			1-OXALIP			2-CTRL			2-OXALIP		
	**INPUT**	19.29	19.64	19.49	19.86	19.72	20.11	19.57	19.39	19.45	19.90	19.80	19.92
	**IP-F**	24.31	24.20	24.52	23.24	23.48	23.70	24.03	23.96	24.12	23.65	23.80	23.72
	**IP-H**	23.99	24.16	24.38	23.53	23.88	23.69	24.67	24.55	24.62	23.97	24.12	23.98
	**IP-K**	23.04	23.18	22.81	24.41	24.36	24.29	22.94	22.88	22.77	24.36	24.33	24.44
	**IP-FLAG**	23.42	23.77	23.58	25.18	25.08	25.29	23.44	23.64	23.89	24.88	24.97	25.02
	**IP-IgG**	36.15	36.23	36.39	36.48	36.73	36.56	36.18	36.26	36.13	36.39	36.56	36.42
		3-CTRL			3-OXALIP			4-CTRL			4-OXALIP		
	**INPUT**	19.61	19.69	19.74	19.95	19.71	19.83	19.65	19.54	19.72	20.30	20.21	20.32
	**IP-F**	23.92	24.07	23.90	21.98	22.28	22.17	23.62	23.49	23.38	22.67	22.44	22.52
	**IP-H**	23.98	24.08	24.03	22.16	22.01	21.96	23.76	23.87	23.65	22.65	22.54	22.77
	**IP-K**	21.10	21.03	20.95	22.30	22.41	22.16	20.97	20.92	20.49	22.70	22.48	22.57
	**IP-FLAG**	22.94	23.10	23.02	24.12	24.26	24.18	23.33	23.22	23.44	24.82	25.02	24.90
	**IP-IgG**	36.62	36.50	36.58	36.52	36.61	36.48	37.29	37.02	37.14	37.57	37.68	37.83

**FOLD CHANGE**	Test-1	Test-2	Test-3	Test-4
**IP-F**	2.45	1.64	3.92	3.02
**IP-H**	1.87	1.99	4.40	3.36
**IP-K**	-1.89	-2.16	-2.16	-2.22
**IP-FLAG**	-2.25	-1.86	-2.02	-1.92
**IP-IgG**	1.06	1.10	1.13	1.07

**IP-Assay # 1**	**Ct SYBR**	**NO-CROSSLINKED** samples. cDNA-Primer 3-REV, PCR-Primers 3-REV + 8-FWD											
		1-CTRL			1-OXALIP			2-CTRL			2-OXALIP		
	**INPUT**	24.51	24.38	24.32	24.43	24.32	24.51	24.04	24.22	23.85	24.31	24.45	24.63
	**IP-F**	26.11	25.84	26.08	24.84	24.79	24.82	25.91	25.86	25.69	24.60	24.48	24.79
	**IP-H**	24.38	24.41	24.24	23.15	23.34	23.49	24.23	24.31	24.14	23.12	23.32	23.44
	**IP-K**	22.25	22.20	22.04	23.19	23.24	23.29	22.26	22.63	22.45	23.62	24.13	23.75
	**IP-FLAG**	22.16	22.21	22.28	23.45	23.23	23.32	22.54	22.45	22.72	23.92	23.86	24.14
	**IP-IgG**	35.77	35.43	35.59	35.18	35.52	35.37	37.18	36.80	36.71	36.94	37.12	37.22
		3-CTRL			3-OXALIP			4-CTRL			4-OXALIP		
	**INPUT**	23.49	23.56	23.38	23.59	23.41	23.28	23.36	23.07	23.30	23.75	23.81	24.04
	**IP-F**	25.63	25.76	24.84	24.54	24.26	24.38	24.69	25.32	25.25	24.36	24.09	24.22
	**IP-H**	23.85	23.98	23.75	22.82	22.89	23.14	23.56	23.39	23.24	22.65	23.28	23.27
	**IP-K**	21.62	21.50	21.42	22.70	22.91	22.55	21.13	21.31	21.02	22.61	22.72	22.67
	**IP-FLAG**	22.55	22.79	22.36	23.48	23.84	23.67	22.01	22.12	22.04	23.74	23.62	23.49
	**IP-IgG**	36.05	36.23	35.80	36.10	35.73	35.87	36.28	36.33	35.82	36.51	36.90	36.73

**FOLD CHANGE**	Test-1	Test-2	Test-3	Test-4
**IP-F**	2.31	3.08	1.95	2.80
**IP-H**	2.05	2.57	1.84	1.94
**IP-K**	-2.08	-1.95	-2.39	-1.85
**IP-FLAG**	-2.14	-1.97	-2.21	-1.91
**IP-IgG**	1.19	1.17	1.05	1.04

IP-Assay # 2	Ct SYBR	**CROSSLINKED samples**. cDNA-Primer 1-REV, PCR-Primers 1-REV + 8-FWD											
		1-CTRL			1-OXALIP			2-CTRL			2-OXALIP		
	**INPUT**	26.89	27.12	26.93	26.27	26.52	26.36	26.91	26.85	27.12	26.62	26.79	26.67
	**IP-F**	27.52	27.57	27.64	26.08	26.17	25.91	27.84	27.90	28.02	26.60	26.71	26.55
	**IP-H**	29.84	29.92	29.97	28.05	28.14	27.88	30.31	30.22	30.35	29.11	28.93	29.21
	**IP-K**	27.93	27.88	28.11	28.81	28.85	28.90	28.31	28.40	28.19	29.09	28.84	28.88
	**IP-IgG**	36.34	36.63	36.77	35.92	35.78	35.98	35.91	36.06	36.14	35.84	35.72	35.66
		3-CTRL			3-OXALIP			4-CTRL			4-OXALIP		
	**INPUT**	23.80	23.91	23.84	22.76	22.79	22.68	23.79	23.74	23.83	22.78	22.64	22.69
	**IP-F**	24.85	24.92	25.03	22.28	22.21	22.34	24.84	24.75	24.89	22.31	22.23	22.14
	**IP-H**	25.12	24.91	24.99	22.68	22.80	22.72	25.32	25.12	25.26	22.85	22.87	22.73
	**IP-K**	21.52	21.39	22.46	21.77	21.87	21.71	22.46	22.33	22.59	22.01	22.08	21.96
	**IP-IgG**	36.86	36.89	36.77	35.48	35.61	35.66	36.91	36.82	36.83	35.84	35.81	35.72

**FOLD CHANGE**				
	Test-1	Test-2	Test-3	Test-4
**IP-F**	1.90	2.05	2.93	2.86
**IP-H**	2.44	1.92	2.24	2.52
**IP-K**	-2.79	-1.87	-2.14	-1.56
**IP-IgG**	1.06	1.02	1.11	0.99

IP-Assay # 2	Ct SYBR	**NO-CROSSLINKED** samples. cDNA-Primer 1-REV, PCR-Primers 1-REV + 8-FWD											
		1-CTRL			1-OXALIP			2-CTRL			2-OXALIP		
	**INPUT**	30.28	29.68	29.90	29.38	29.60	29.52	29.45	29.35	29.52	29.41	29.35	29.49
	**IP-F**	30.32	30.49	30.36	28.93	29.08	28.94	30.31	30.35	30.21	29.14	29.25	29.37
	**IP-H**	30.49	30.38	30.56	28.85	28.72	28.91	30.27	30.41	30.38	29.38	29.22	29.41
	**IP-K**	27.69	27.89	27.71	28.52	28.49	28.69	27.98	28.12	28.05	28.88	29.08	28.96
	**IP-IgG**	36.19	36.38	36.24	35.98	36.20	35.49	36.37	36.41	36.45	36.44	36.38	36.51
		3-CTRL			3-OXALIP			4-CTRL			4-OXALIP		
	**INPUT**	30.32	30.19	30.31	30.19	29.94	30.12	30.44	30.61	30.54	29.85	29.77	29.79
	**IP-F**	30.46	30.53	30.42	29.81	28.89	29.34	30.37	30.46	30.32	28.65	28.74	28.63
	**IP-H**	30.70	30.51	30.64	28.81	29.13	29.08	30.40	30.45	30.54	28.71	28.63	28.68
	**IP-K**	28.64	28.49	28.61	29.32	29.21	29.26	28.79	28.69	28.74	28.92	29.08	28.89
	**IP-IgG**	36.67	36.74	36.72	36.20	36.38	36.29	36.48	36.61	36.54	35.78	35.82	35.88

**FOLD CHANGE**				
	Test-1	Test-2	Test-3	Test-4
**IP-F**	1.94	2.02	1.91	1.98
**IP-H**	2.30	1.99	2.68	2.09
**IP-K**	-2.38	-1.93	-1.83	-1.93
**IP-IgG**	-1.05	-1.04	1.17	-1.01

**IP-Assay # 3**	**Ct SYBR**	NO-CROSSLINKED samples. cDNA-Primer 3-REV, PCR-Primers 3-REV + 8-FWD											
		1-CTRL			1-OXALIP			2-CTRL			2-OXALIP		
	**INPUT**	28.25	28.22	28.11	29.13	29.18	29.11	28.24	28.18	28.25	29.14	29.16	29.27
	**IP-F**	28.10	28.42	28.32	28.10	28.16	28.23	28.06	27.96	28.14	27.97	27.74	27.91
	**IP-K**	28.20	28.08	28.15	30.21	30.24	30.38	28.37	28.42	28.31	30.53	30.40	30.57
	**IP-FLAG**	27.25	27.34	27.23	30.27	30.38	30.45	28.21	28.02	28.12	31.08	31.11	31.21
		3-CTRL			3-OXALIP			4-CTRL			4-OXALIP		
	**INPUT**	26.47	26.32	26.44	27.12	27.20	27.16	27.59	27.68	27.54	27.34	27.25	27.23
	**IP-F**	26.55	26.64	26.47	26.10	26.25	25.27	28.39	28.30	28.36	27.09	26.95	26.98
	**IP-K**	26.97	27.11	27.15	28.89	28.81	28.76	26.40	26.31	26.26	27.16	27.24	27.11
	**IP-FLAG**	28.14	27.96	28.04	30.58	30.72	30.68	28.95	29.07	28.89	30.84	30.85	31.02
**FOLD CHANGE**													
	Test-1	Test-2	Test-3	Test-4									
**IP-F**	2.09	2.21	2.69	2.02									
**IP-K**	-2.28	-2.24	-1.99	-2.28									
**IP-FLAG**	-4.43	-4.14	-3.64	-2.28									

**IP-Assay # 4**	**Ct SYBR**	NO-CROSSLINKED samples. cDNA-Primer 3-REV, PCR-Primers 3-REV + 8-FWD											
		1-CTRL			1-OXALIP			2-CTRL			2-OXALIP		
	**INPUT**	25.23	25.31	25.35	25.51	25.75	25.60	24.89	25.10	24.84	25.42	25.34	25.32
	**IP-F**	24.52	24.41	24.36	22.85	22.97	23.08	24.47	24.32	24.36	23.48	23.61	23.39
	**IP-K**	20.17	20.21	20.38	21.68	21.76	21.82	19.79	19.92	19.73	21.74	21.84	21.69
	**IP-FLAG**	22.45	22.56	22.29	25.03	24.86	24.81	22.16	22.09	22.28	24.78	24.89	24.84
		3-CTRL			3-OXALIP								
	**INPUT**	26.34	26.53	26.42	27.31	27.20	27.18						
	**IP-F**	27.61	27.47	27.73	26.98	27.08	26.91						
	**IP-K**	25.96	25.78	25.88	28.30	28.11	28.15						
	**IP-FLAG**	26.44	26.32	26.57	29.38	29.33	29.17						
**FOLD CHANGE**													
	Test-1	Test-2	Test-3										
**IP-F**	3.45	2.47	2.66										
**IP-K**	-2.26	-2.88	-2.85										
**IP-FLAG**	-4.42	-4.75	-4.14										

**IP-Assay # 5**	**Ct SYBR**	NO-CROSSLINKED samples. cDNA-Primer 3-REV, PCR-Primers 3-REV + 8-FWD											
		1-CTRL			1-OXALIP			2-CTRL			2-OXALIP		
	**INPUT**	22.21	22.38	22.25	22.56	22.45	22.41	23.36	22.28	22.42	23.41	23.22	23.33
	**IP-F**	21.42	21.33	21.56	20.29	20.48	20.36	20.62	20.76	20.69	20.48	20.34	20.28
	**IP-FLAG**	20.77	20.84	21.02	21.98	22.15	22.09	21.10	20.91	21.04	22.71	22.62	22.57
		3-CTRL			3-OXALIP			4-CTRL			4-OXALIP		
	**INPUT**	24.08	24.26	24.14	24.14	23.94	24.06	24.04	24.19	24.14	24.06	24.10	23.94
	**IP-F**	23.56	23.67	23.69	22.11	22.23	22.02	24.77	24.96	24.92	23.61	23.42	23.45
	**IP-FLAG**	24.18	24.10	23.96	24.96	24.88	25.04	22.58	22.50	22.38	23.52	23.30	23.44
**FOLD CHANGE**													
	Test-1	Test-2	Test-3	Test-4									
**IP-F**	2.38	1.94	2.65	2.46									
**IP-FLAG**	-2.00	-1.98	-1.99	-2.03									

IP-Assay # 6 **Ct SYBR** NO-CROSSLINKED samples. cDNA-Primer 3-REV, PCR-Primers 3-REV + 8-FWD

	1-CTRL			1-OXALIP			2-CTRL			2-OXALIP		
**INPUT**	26.55	26.34	26.41	25.41	25.52	25.36	26.75	26.83	25.67	25.65	25.60	25.69
**IP-F**	29.15	29.22	29.37	26.87	26.84	26.66	28.63	28.49	28.55	26.36	26.24	26.28
**IP-H**	28.28	28.04	28.26	25.83	25.78	25.94	28.58	28.37	28.48	26.56	26.74	26.58
**IP-K**	26.44	26.39	26.55	26.81	26.62	26.75	26.17	26.27	26.38	26.91	26.79	26.76

	3-CTRL			3-OXALIP			4-CTRL			4-OXALIP		
**INPUT**	24.29	24.56	24.51	25.08	24.91	24.94	24.53	24.39	24.34	25.95	25.86	25.82
**IP-F**	27.66	27.45	27.52	27.15	27.23	27.09	27.21	27.05	27.15	27.72	27.56	27.61
**IP-H**	28.72	28.81	28.85	27.86	28.05	27.91	28.52	28.66	28.61	28.29	28.31	28.40
**IP-K**	26.85	26.77	26.92	28.50	28.64	28.55	26.06	26.25	26.14	28.86	28.93	28.78

FOLD CHANGE

	Test-1	Test-2	Test-3	Test-4
**IP-F**	2.74	2.82	1.88	1.95
**IP-H**	2.53	2.11	2.60	3.29
**IP-K**	-2.38	-2.47	-2.29	-2.38

IP-Assay # 7 **Ct SYBR** NO-CROSSLINKED samples. cDNA-Primer 3-REV, PCR-Primers 3-REV + 8-FWD

	1-CTRL			1-OXALIP			2-CTRL			2-OXALIP		
**INPUT**	26.32	26.19	26.24	25.09	25.31	25.15	26.02	25.92	26.09	25.32	25.14	25.21
**IP-F**	31.14	31.17	31.25	28.66	28.69	28.86	31.02	30.85	30.90	28.92	28.77	28.98
**IP-H**	30.84	30.93	31.06	28.78	28.80	29.02	31.43	31.35	31.58	29.56	29.72	29.48
**IP-K**	23.48	23.59	23.43	23.45	23.63	23.61	23.23	23.06	23.18	23.32	23.36	23.49

	3-CTRL			3-OXALIP			4-CTRL			4-OXALIP		
**INPUT**	28.09	27.96	28.18	27.48	27.36	27.39	28.26	28.17	28.34	27.77	27.86	27.71
**IP-F**	33.11	32.94	32.93	31.27	31.32	31.18	32.63	32.72	32.78	31.36	31.19	31.24
**IP-H**	33.21	33.11	33.04	31.34	31.55	31.60	32.55	32.70	32.68	31.01	31.15	30.90
**IP-K**	25.82	25.64	25.76	26.38	26.45	26.28	26.91	26.86	26.77	27.47	27.46	27.65

FOLD CHANGE

	Test-1	Test-2	Test-3	Test-4
**IP-F**	2.61	2.37	2.10	1.96
**IP-H**	2.01	2.11	1.94	2.21
**IP-K**	-2.19	-2.03	-2.46	-2.23

IP-Assay # 8 **Ct SYBR** NO-CROSSLINKED samples. cDNA-Primer 3-REV, PCR-Primers 3-REV + 8-FWD

		1-CTRL			1-OXALIP			2-CTRL			2-OXALIP	
**INPUT**	28.18	27.98	28.20	28.08	28.24	28.13	28.16	28.21	27.99	28.31	28.33	28.19
**IP-F**	31.70	31.56	31.61	30.32	30.43	30.48	31.57	31.46	31.60	30.34	30.42	30.33
**IP-H**	31.63	31.79	31.72	30.71	30.85	30.73	31.88	31.86	32.02	31.11	31.02	31.21
**IP-K**	26.23	26.20	26.38	27.18	27.32	27.34	26.31	26.17	26.19	27.54	27.36	27.42

		3-CTRL			3-OXALIP			4-CTRL			4-OXALIP	
**INPUT**	27.52	27.61	27.65	28.02	27.98	28.06	27.40	27.32	27.28	27.78	27.91	27.83
**IP-F**	31.92	31.98	31.86	31.44	31.38	31.29	31.69	31.84	31.85	31.36	31.51	31.46
**IP-H**	31.82	31.71	31.78	31.11	31.15	31.28	31.95	31.88	32.04	31.54	31.57	31.46
**IP-K**	27.64	27.56	27.55	28.92	29.03	28.87	28.26	28.30	28.18	29.72	29.82	29.78

FOLD CHANGE

	Test-1	Test-2	Test-3	Test-4
**IP-F**	2.37	2.53	1.97	1.82
**IP-H**	1.97	1.95	2.02	1.92
**IP-K**	-1.97	-2.08	-1.91	-2.03

Table S2 (related to Figure 6). Splicing units responsive to oxaliplatin. Oxaliplatin responsive units are defined as displaying a ΔPSI greater than 5 percentage points and a *P* value < 0.05. Units which oxaliplatin-induced shift is compromised by the siRNA-mediated depletion of SRSF10 (annotated as ^{si}) with greater than 5 percentage points (*P* value < 0.05) are highlighted. The size of long (L) and small (S) fragments amplified by RT-PCR is indicated.

GENE	Variant size (bps)		Primer Sequence		CTRL-OXALI		CTRL^{si}-OXALI^{si}		OXALI-OXALI^{si}		CTRL-CTRL^{si}	
	S	L		Reverse	Δ PSI	*P value*	Δ PSI	*P value*	Δ PSI	*P value*	Δ PSI	*P value*
TNFRSF10B	584	671	CAGGACTATAGCACTCACTG	CTCCTCCTCTGAGACCTTT	-21.6	0.0001	-15.12	0.00019	7.5	0.00009	1.0	0.6185
GTF2H2	152	250	GTTGAATTGCCCTGCCTG	GTTCTTTCATAGCCTCCTTCCC	-7.3	0.0012	-6.70	0.00437	5.1	0.00315	4.5	0.0208
CASP8	433	478	CACTAGAAAGGAGGAGATGG	GATGATCAGACAGTATCCCC	23.5	0.0000	20.80	0.00010	-5.3	0.01684	-2.6	0.0211
CDC25B	101	224	GAGCAGTTTGCCATCAGACG	TCTGCCAGAGCATGGGTG	6.7	0.0026	6.45	0.00031	-5.3	0.00920	-5.0	0.0000
DOM3Z	241	314	ATAGTGACGTGGCGGGGGCA	GAGAAGAGCAGAGGGTGGCTTC	62.2	0.0000	53.84	0.00001	-6.3	0.01850	2.1	0.2562
NAP1L4	341	379	CGAGGAGGGAGAAGACGAGGAT	GAAGTCCAGAGCTACAGGCAC	13.9	0.0000	12.22	0.00237	-8.8	0.00056	-7.1	0.0137
SPTAN1	308	368	GGTGGAAAGTGGAAGTGAACGA	AACCTGCTCCAAGTCTGCTCC	15.5	0.0001	10.75	0.00200	-9.2	0.00417	-4.5	0.0051
PCBP4	188	317	GCAAATGGTGGAAATGTCTCCAG	TGGTTGGCAGAGAGAAGAACAG	7.8	0.0158	2.69	0.41378	-10.2	0.00866	-5.1	0.1452
BRCA1	188	311	AACCAGTCTCAGTGTCCAACTC	CACGCTTCTCAGTGGTGTTCAA	24.0	0.0000	0.08	0.64782	-13.1	0.00006	-2.4	0.0138
CHEK2	194	256	CAGCTCTCAATGTTGAAACAGAA	TCTGGCTTTAAGTCACGGTGT	43.0	0.0001	28.44	0.00002	-13.7	0.01041	0.9	0.2252
BCL2L1	267	456	ATGGCAGCAGTAAAGCAAGCG	TCATTTCCGACTGAAGAGTGA	41.1	0.0007	29.93	0.00009	-15.9	0.02520	-4.8	0.0291
MLH3	153	225	CCCTATCGTTTCACCAAAGG	TTTCCGACCAGAGCCTTGT	18.1	0.0096	2.68	0.38760	-17.4	0.01187	-2.0	0.5025
RBBP8	145	226	AATGTGCCTCTCGCCTTACC	TGCTCCACACTTCTACTTGCTT	24.0	0.0001	13.68	0.00017	-17.5	0.00020	-7.1	0.0027
BCLAF1-2	479	626	GTACCCTGAGGAAGCATAC	AGTACCACGACCTCTTCCT	15.7	0.0001	1.11	0.34443	6.2	0.03993	15.3	0.0071
CHEK1	103	327	GACTGGGACTTGGTGCAAAC	TGCCATGAGTTGATGGAAGA	-5.2	0.0168	-5.75	0.00309	-9.5	0.00067	-8.9	0.0021
CASP9	360	810	TCCTGCTTAGAGGACACAG	GGACACAAAGATGTCACTGG	-14.5	0.0201	-13.53	0.05418	-13.9	0.04027	-14.9	0.0262
MCL1	134	382	CCAAGGACACAAAGCCAATG	TGGAAGAACTCCACAAACCC	-8.5	0.0002	-9.04	0.00058	3.5	0.00068	4.0	0.0183
AURKA	119	217	CCAGAGTGCGGGGATATTTGAT	TGCAGTTTTCTTTAGATCGGTCC	-7.0	0.0005	-6.63	0.00065	1.9	0.00532	1.6	0.1541
EXO1	193	298	ACTATCGCACTCAGCCATTCTT	TGTAGCAATCCCTGTATCCCCA	-5.9	0.0012	-10.03	0.00016	-2.4	0.02713	1.7	0.0770
CDC25A	145	265	CGACCCAGATGAGAACAAGG	ATCGAGAAGGTCCACGAAGC	5.1	0.0087	1.41	0.04490	-3.0	0.03444	0.7	0.3692
CASC4	241	409	CAACTGGACAACCTCTCTC	GCAGGATCCATTTGAAGCTC	7.1	0.0018	6.61	0.00146	-3.4	0.00943	-2.9	0.0520
AKIP1	252	333	CTCTAGAAGTGCTGGAGAG	GACCATTCCCTATGTCCAAG	7.5	0.0023	8.31	0.00517	-4.1	0.03329	-4.9	0.0200
ARID4B	625	883	GAGGAGTACTGTAGATCAGC	TGGCATCAGTGAGATCCAG	7.7	0.0257	-7.26	0.04465	-4.8	0.04166	10.2	0.0466
ESYT2	124	187	TCAAAGCTGACAAAGACCAAGC	TGGACAACAGGATTTGGGTTGC	14.7	0.0002	11.62	0.00050	-2.2	0.15544	1.0	0.4191
RBM41	*94*	*194*	TTTCAGGGAGCTGATCGTCACT	AGATCTCTTTCAGTCACCCGAG	10.2	0.0165	5.41	0.09282	1.0	0.21466	5.8	0.1725
AXIN1	535	643	GAGGCTACTCAGAGAGTGTT	CAGAAGTAGTACGCCACAAC	5.8	0.0145	7.07	0.06774	-1.2	0.53255	-2.4	0.4084
SDCCAG8	180	417	GTGCCTGAGACTAACAGAAC	ATCGTCTCATGTACTCTCCC	21.1	0.0006	19.12	0.00125	0.6	0.79139	2.6	0.3380
FASTK	410	730	CTGCTCTCTGCTCAGACCT	GGACCAACTTCTGTACCAC	6.4	0.0012	11.10	0.00061	0.1	0.96410	-4.6	0.0008

Table S3 (related to Figure 6). Splicing units non-responsive to oxaliplatin. Splicing units responsive to siSRSF10 (annotated as ^{si}) with ΔPSI CTRL-CTRL^{si} >5% and *P* values < 0.05 are highlighted.

GENE	Variant size (bps)		Primer Sequence		CTRL-OXALI		CTRL-CTRL^{si}	
	S	L	Forward	Reverse	Δ PSI	*P value*	Δ PSI	*P value*
BCLAF1	160	679	CACCACAGAATGCTCCAAGA	GGGCTTTCCTCTCTGAAGGT	1.64	0.8317	57.4	0.000013
AKIP1	162	243	GTTATCACAGAGGCGAGTC	CAACTGCTACCACATGAGTC	-3.07	0.147785	18.74	0.004313
RAD17	154	338	GCAAGTCCTAAACTACGGATGGG	TGTGGCAGTAATAGTAGAGACGC	-0.40	0.807426	-3.14	0.013881
BRCA1	103	177	GCTAGAAATCTGTTGCTATGGGC	TGGAAGCCATTGTCCTCTGTCC	0.48	0.380386	-1.53	0.023523
BNIP1	339	468	CCGGATCTGTAACCAAGAG	CTTAAGAGATCTCCTCCCTG	0.18	0.705796	-1.72	0.035155
XRCC3	103	140	GGAGAAGGCCGAGAGGAGCA	AACGGCAGTCCGGCTCCTGA	-5.04	0.296109	-4.58	0.051925
SEMA6A	381	546	GAGCAAGCAGCTCTCTGTAT	GATCACAGACGCAGTAGAC	-0.99	0.183298	1.44	0.064269
RAD1	272	381	TCTGACCCAACAGATCCAAGAC	GTCACCACTCCTCCTTCTTCCA	0.84	0.671675	3.87	0.086792
GDPD1	1015	1293	GCCACTACGTCATCTACAG	CAGAGAACTCTCATCCAGG	4.57	0.070515	4.33	0.103870
ATRIP	316	397	TAGCGTGCTGCTGGCTGTT	CTTCTGCGATAGGCCGTG	1.03	0.123691	1.03	0.104551
TRAF2	684	768	GTCTGTCCCAGTGATGGAT	GTACCTGCTGGTGTAGAAG	0.00	#DIV/0!	0.70	0.116584
FYN	464	620	CAACTGGAGAGACAGGTTAC	CTCAGACACCACTGCATAG	-0.22	0.463654	-0.40	0.129787
RAD50	104	169	GCAATTTCGTGATGTCAATGG	CATTTCTCGGTCAATTTCTGC	0.16	0.596823	-0.47	0.141932
AURKB	199	272	CGGGGCGGGAGATTTGAAAAGT	TGTGGGCTGGACATTGGAGC	-6.62	0.179320	-5.83	0.169429
RAD51	220	342	GCCACCGCCCTTTACAGAACA	GGCATCTCCCACTCCATCTGC	1.84	0.160187	-0.60	0.215544
CDK2	219	297	GGTGGAAAAGATCGGAGAGGGC	TTGATGAGGGGAAGAGGAATGC	0.62	0.084037	0.40	0.216825
ERCC1	104	176	GCCTATGAGCAGAAACCAGCG	GCCAGATCTTCTCTTGATGCGG	2.19	0.079112	-1.51	0.252307
RELA	576	908	AGAGGAGCACAGATACCAC	GGATGACGTAAAGGGATAGG	-6.43	0.161509	-5.45	0.253329
APAF1	768	897	GGACCCTCAAGAGGATATG	GAAAGTACTGTACCCTGGTG	4.51	0.396638	6.69	0.261705
CASP7	512	546	GACCGAGTGCCTACATATC	GCTCCTCCACGAGTAATAG	-0.35	0.309065	-0.36	0.308362
APEX1	168	243	GAGCTGTCGGAGGTGCTGGT	CCTCCTTAGAGACGTTCTGAGC	6.36	0.167711	4.28	0.317938
MPG	111	304	GTCCGAGTCCCACGAAGC	CTGCCCTGCTCTAGCTGGT	-0.80	0.375239	-0.91	0.350710
MUTYH	107	257	CTGTCTTCATCAGCGTGGGC	CATGCTTCTGCCTCCCTTCCTG	2.97	0.314080	-2.61	0.370626
PRKDC	210	303	CCATGACGAGAGGGAACACCC	AGCCAATCTTTATATTCACACGGC	0.32	0.173385	-0.19	0.373901
MDM2	1016	1181	GTGCCAAGCTTCTCTGTGA	GGTTGTCTACATACTGGGC	2.75	0.373901	2.75	0.373901
CHEK2	231	360	TACCAGCACGATGCCAAACTCC	TTGTCCCTCCCAAACCAGTAGT	-0.23	0.148132	-0.19	0.388938
MID1	104	257	AGAACTGGAGACCCTTTTGGCT	TGAGAAGATCACACTCCTCTGTCA	-0.07	0.670527	-0.18	0.447287
MERTK	298	384	CTCGGATCTCTGTTCAAGTC	GTAGTTCCTCACTGACTCC	0.91	0.595893	0.46	0.578754
BCL2L12	187	330	TCCTAGCTGCCTTCCTTAG	GTATGGCTTCCTTCTCTGTC	5.49	0.204395	1.96	0.597670
MAPK1	438	570	CTACTTCCTCTACCAGATCC	TAGTACTGCTCCAGGTAGG	1.41	0.052545	-0.43	0.624316
PRKDC	305	398	CCATTGCCAGAGTACCACGT	AGCCAATCTTTATATTCACACGGC	0.36	0.150542	0.13	0.626807
CDK1	110	281	TGGATTCTATCCCTCCTGGTCA	TGGAGTTGAGTAACGAGCTGAC	0.24	0.574070	0.17	0.647727
FAS	162	409	GAGACTCAGAACTTGGAAGG	GAAGAAGACAAAGCCACCC	-1.04	0.165357	0.25	0.856845
BCL2L11	130	400	TACCTCCCTACAGACAGAG	CCTCCTTGCATAGTAAGCGT	-8.51	0.081708	-0.73	0.858097
ATRIP	157	242	CTTCCTGTGATTTCTTGCCC	GAGTGGGAACAGAGCTGAGG	-2.68	0.066094	-0.23	0.888742
SUMO1	216	291	GGAAGTGACGCGAGGCGTAG	CTTTGAGTTTCTTGAGATGTGTTGTCA	0.84	0.068810	0.01	0.959054
C16orf62	489	574	CTCAGACCAGAAAGTGAAGG	GTACAAGGGGAGGTAAGATG	-0.06	0.878093	0.01	0.983024
CDKN1A	243	384	TGCCGAAGTCAGTTCCTTGTGG	TCGGTGACAAAGTCGAAGTTCC	-0.32	0.373901	0.00	#DIV/0!
MID1	213	313	GATCCCAAATCTGCTCATCG	TTTCCTTGCTATTGTGTCTCACC	0.00	#DIV/0!	0.00	#DIV/0!
MLH1	102	194	AGATGGAAAACTGAAAGCCCCT	TTTTAACTGAGAAACTAATGCCTGCA	0.00	#DIV/0!	0.00	#DIV/0!

Table S4 (related to Figure 6). Compilation of RT-qPCR values obtained for immunoprecipitation of *Bcl-x*, *BCLAF1* and *AKIP1* pre-mRNA with antibodies targeting FLAG-SRSF10. The anti-Flag antibody was used to recover FLAG-SRSF10. RT-qPCR was performed on the same amount of RNA for input and immunoprecipitated material from both conditions (oxaliplatin-treated and untreated in three biological replicates). Ct values for all tests are indicated. After calculating the ΔCt for each pair of IP/input, ΔΔCt values were calculated for oxaliplatin-treated/untreated (CTRL) pair, and their averages were used to obtain a fold-difference value, as described in Experimental Procedures. The position of the primers used for monitoring pre-mRNA recovery is indicated on a diagram for each unit.

BCLAF1 **Ct SYBR** cDNA-Primer Random Hexamer, PCR-Primers BCLAF1-1-FWD +BCLAF1-2-REV

	1-CTRL			1-OXALIP			2-CTRL			2-OXALIP			3-CTRL			3-OXALIP		
INPUT	31.3	31.6	31.3	31.5	31.6	31.9	31.9	31.8	31.6	31.3	31.9	31.6	30.6	30.9	30.7	32.6	32.8	31.8
IP-FLAG	26.4	26.3	26.4	27.1	27.1	27.0	26.1	26.0	25.9	26.8	26.7	26.8	26.5	26.3	26.1	27.4	27.2	27.0

FOLD CHANGE

	Test-1	Test-2	Test-3
IP-FLAG	-1.34	-1.93	1.75

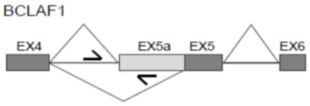

AKIP1 **Ct SYBR** cDNA-Primer Random Hexamer, PCR-Primers AKIP1-1-FWD +AKIP1-2-REV

	1-CTRL			1-OXALIP			2-CTRL			2-OXALIP			3-CTRL			3-OXALIP		
INPUT	32.6	33.2	32.1	33.7	32.4	32.7	32.9	34.0	34.3	33.6	33.5	33.3	32.6	32.1	32.3	33.8	33.6	33.5
IP-FLAG	30.8	30.7	31.0	32.7	31.7	31.9	31.1	31.4	30.7	32.0	31.3	31.9	30.6	31.0	31.0	32.0	31.7	32.9

FOLD CHANGE

	Test-1	Test-2	Test-3
IP-FLAG	-1.91	-1.90	-1.04

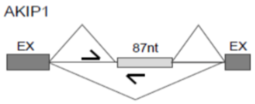

BCL-X **Ct SYBR** cDNA-Primer andom Hexamer, PCR-Primers 3-REV + 8-FWD

	1-CTRL			1-OXALIP			2-CTRL			2-OXALIP			3-CTRL			3-OXALIP		
INPUT	30.9	31.1	30.5	33.5	33.5	33.0	31.9	31.9	32.2	33.5	33.3	34.1	31.1	31.6	32.2	34.1	34.6	33.8
IP-FLAG	24.2	24.1	24.1	31.0	31.4	31.9	24.7	24.6	24.8	30.6	30.9	30.9	24.4	24.7	24.4	31.2	30.9	31.1

FOLD CHANGE

	Test-1	Test-2	Test-3
IP-FLAG	-27.34	-22.80	-17.10

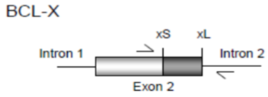

Table S5 (related to Figure 3). Sequence of primers used for mutating S133 and S131 in SRSF10 and deleting or producing different domains of SRSF10

Δ-131-133-FWD	5'- TCA AGA CGG TTT GAT TAC AAC TAT AGA AGA TC -3'
Δ-131-133-REV	5'- ATC AAA CCG TCT TGA TCT CCT CCT TTC ATA ACT -3'
SRSF10-ALA-FWD	5'- GCT CGG GCT TTT GAT TAC AAC TAT AGA AGA -3'
SRSF10-ALA-REV	5'- AGC CCG AGC TCT TGA TCT CCT CCT TTC ATA A -3'
SRSF10-NO-S133-FWD	5'- AGT CGG TTT GAT TAC AAC TAT AGA AGA TCG -3'
SRSF10-NO-S133-REV	5'- ATC AAA CCG ACT TCT TGA TCT CCT CCT TTC -3'
SRSF10-NO-S131-FWD	5'- TCA AGA CGG TCT TTT GAT TAC AAC TAT AGA -3'
SRSF10-NO-S131-REV	5'- AGA CCG TCT TGA TCT CCT CCT TTC A -3'
BAMHI-SRSF10-FWD	5'- CTT GGA TCC ATG TCC CGC TAC CT -3'
SRSF10-ECORI-REV	5'- TC GAA TTC TCA GTG GCC ACT GGA CTT A -3'
SRSF10-RS2-FWD	5'- CCC CAA CAC GCA TTC CGA CAA TGA TAG ATT C -3'
SRSF10-RS2-REV	5'- GTC GGA ATG CGT GTT GGG GGG ACG CAG GTA G -3'
SRSF10-RS1-FWD	5'- TAG CAG AAG CTG GAC TAG TCC TAA GTC CAG T -3'
SRSF10-RS1-REV	5'- GAC TAG TCC AGC TTC TGC TAC GCC GTG GTC T -3
SRSF10-RMM-FWD	5'- GGA CGG CAG ATT GAA ATA CAG TGG ACT AGT-3'
SRSF10-RMM-REV	5'- ACT GGA CTT AGG ACT CCA CTG TAT TTC -3'
HA-DRRM-FWD	5'- CCA ACA CGT TTG CCC AGG GGG ATC GAA AG -3'

HA-DRRM-REV	5'- CCT GGG CAA ACG TGT TGG GGG GAC GCA -3'
HA-DRS1-FWD	5'- GAC AGA TAC CAT TCC GAC AAT GAT AGA TTC -3'
HA-DRS1-REV	5'- GTC GGA ATG GTA TCT GTC ATA ATC ATC ATA GC -3'
HA-DRS2-FWD	5'- CAC CGA AAT TGG ACT AGT CCT AAG TCC AGT G -3'
HA-DRS2-REV	5'- ACT AGT CCA ATT TCG GTG TTT GAA TCT ATC -3'
HA-DRS-FWD	5'- GAC AGA TAC TGG ACT AGT CCT AAG TCC AGT -3'
HA-DRS-REV	5'- AGG ACT AGT CCA GTA TCT GTC ATA ATC ATC -3'
pcDNA3.1 FWD	5'- AGC AGA GCT CTC TGG CTA ACT AGA GAA CCC -3'
pcDNA3.1 REV	5'- GGC TGG CAA CTA GAA GGC ACA GTC GAG -3'

Table S6 (related to Figure 6). List of primers used to monitor splicing units by RT-PCR

BRCA1-FWD	5'- AACCAGTCTCAGTGTCCAACTC
BRCA1-REV	5'-CACGCTTCTCAGTGGTGTTCAA-3'
CHEK2-FWD	5'-CAGCTCTCAATGTTGAAACAGAA-3'
CHEK2-REV	5'-TCTGGCTTTAAGTCACGGTGT-3'
MLH3-FWD	5'-CCCTATCGTTTCACCAAAGG-3'
MLH3-REV	5'-TTTCCGACCAGAGCCTTGT-3'
RBBP8-FWD	5'-AATGTGCCTCTCGCCTTACC-3'
RBBP8 -REV	5'-TGCTCCACACTTCTACTTGCTT-3'
PCBP4-FWD	5'-GCAAATGGTGGAAATGTCTCCAG-3'
PCBP4-REV	5'-TGGTTGGCAGAGAGAAGAACAG-3'
TNFRSF10B-FWD	5'-CAGGACTATAGCACTCACTG-3'
TNFRSF10B-REV	5'-CTCCTCCTCTGAGACCTTT-3'
CASP8-FWD	5'-CACTAGAAAGGAGGAGATGG-3'
CASP8-REV	5'-GATGATCAGACAGTATCCCC-3'
GTF2H2-FWD	5'-GTTGAATTGCCCTGCCTG-3'
GTF2H2-REV	5'-GTTCTTTCATAGCCTCCTTCCC-3'
CDC25B-FWD	5'-GAGCAGTTTGCCATCAGACG-3'
CDC25B-REV	5'-TCTGCCAGAGCATGGGTG-3'
APAF1-FWD	5'-GGACCCTCAAGAGGATATG-3'
APAF1-REV	5'-GAAAGTACTGTACCCTGGTG-3'
AURKB-FWD	5'-CGGGGCGGGAGATTTGAAAAGT-3'
AURKB -REV	5'-TGTGGGCTGGACATTGGAGC-3'
CDK1-FWD	5'-TGGATTCTATCCCTCCTGGTCA-3'
CDK1-REV	5'-TGGAGTTGAGTAACGAGCTGAC-3'
CDK2-FWD	5'-GGTGGAAAAGATCGGAGAGGGC-3'
CDK2-REV	5'-TTGATGAGGGGAAGAGGAATGC-3'
EXO1-FWD	5'-ACTATCGCACTCAGCCATTCTT-3'
EXO1-REV	5'-TGTAGCAATCCCTGTATCCCCA-3'
FASTK-FWD	5'-CTGCTCTCTGCTCAGACCT-3'
FASTK -REV	5'-GGACCAACTTCTGTACCA-3'C
CDC25A-FWD	5'-CGACCCAGATGAGAACAAGG-3'
CDC25A-REV	5'-ATCGAGAAGGTCCACGAAGC-3'
BAX-FWD	5'-GAGCAGATCATGAAGACAGG-3'
BAX-REV	5'-GTCCCAAAGTAGGAGAGGA-3'
CHEK2-2-FWD	5'-CAAAGTGGTGGGGAATAAACGC-3'
CHEK2-2-REV	5'-CGCCAAGTAGGTGGGGGTTC-3'
RAD17-FWD	5'-CAGTCACAATCTTTTGATGAGGACG-3'
RAD17-REV	5'-TGGTTGCCTTTCTAAAACTTGAGC-3'
BCLAF1-1-FWD	5'- TGAGGGATTGGGCAACACATTT -3
BCLAF1-2-REV	5'- CTCTTTATCCCTGGTATTACCCCTA-3
AKIP1-1-FWD	5'- GCTGACAGAACAATTACTAACTCCT -3'
AKIP1-2-REV	5'- CTCCAGGACCAAGGGATGTC -3'

Supplemental Experimental Procedures

Gel-shift assay

The pre-mRNA substrate of *Bcl-x* was synthesized in vitro using T3 RNA polymerase in the presence of a *Bcl-x* PCR-produced template made with primers T3-Bcl-x-FWD 5'-TAACCCTCACTAAAGCATATCAGAGCTTTG-3', Bcl-xL-REV 5'- GGGCTTGGTTCTTAC-3', a transcription mix and minimal amounts of ^{32}P-UTP. The uniformly radiolabeled RNA was purified in a denaturing gel. The purified RNA (5000 cpm) was incubated on ice for 15 min in splicing conditions and in the presence or the absence of 2.5 µM of recombinant hnRNP F or SRSF10 proteins prior to the addition of heparin to a final concentration of 0.75 mg/mL and loading dye. The reactions were incubated for another 3 min on ice before loading the samples on a 5% native acrylamide gel (29:1, acrylamide:bisacrylamide, 5% glycerol, 50 mM Tris pH 8.8, 50 mM glycine) in Tris-glycine running buffer (50 mM Tris pH 8.8, 50 mM glycine). After 2 hours at 150 V, the gel was exposed on a film for visualization.

Supplemental References

Cox, J., and Mann, M. (2008). MaxQuant enables high peptide identification rates, individualized p.p.b.-range mass accuracies and proteome-wide protein quantification. Nature biotechnology *26*, 1367-1372.

Paz, I., Kosti, I., Ares, M., Jr., Cline, M., and Mandel-Gutfreund, Y. (2014). RBPmap: a web server for mapping binding sites of RNA-binding proteins. Nucleic acids research *42*, W361-367.

A

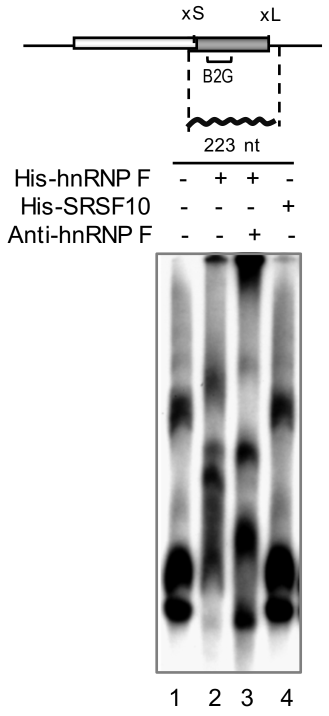

B

SB1

xS splice site

B2G

CTTCAGAATCTTATCTTGGCTTTGGATCTTAGAAGAGAATCACTAACCAGAGACGAGACTCAGTGAGTGA
GCAGGTGTTTTGGACAATGGACTGGTTGAGCCCATCCCTATTATAAAAATGTCTCAGAGCAACCGGGAG
CTGGTGGTTGACTTTCTCTCCTACAAGCTTTCCCAGAAAGGATACAGCTGGAGTCAGTTTAGTGATGTG
GAAGAGAACAGGACTGAGGCCCCAGAAGGGACTGAATCGGAGATGGAGACCCCCAGTGCCATCAATG
GCAACCCATCCTGGCACCTGGCAGACAGCCCCGCGGTGAATGGAGCCACTGGCCACAGCAGCAGTTT
GGATGCCCGGGAGGTGATCCCCATGGCAGCAGTAAAGCAAGCGCTGAGGGAGGCAGGCGACGAGTTT
GAACTGCGGTACCGGCGGGCATTCAGTGACCTGACATCCCAGCTCCACATCACCCCAGGGACAGCATA
TCAGAGCTTTGAACAGGTAGTGAATGAACTCTTCCGGGATGGGGTAAACTGGGGTCGCATTGTGGCCTT
TTTCTCCTTCGGCGGGGCACTGTGCGTGGAAAGCGTAGACAAGGAGATGCAGGTATTGGTGAGTCGGA
TCGCAGCTTGGATGGCCACTTACCTGAATGACCACCTAGAGCCTTGGATCCAGGAGAACGGCGGCTGG
gtaagaaccaagccccttgtgtgtcccttttctttggcctctggtcagagatccccaacagcccttcttctgtatctctttctgttgtgttgggtgattgt

putative high SRSF10 binding sites

putative medium SRSF10 binding sites

Supplemental Figure 1

Shkreta_Suppl-Fig2

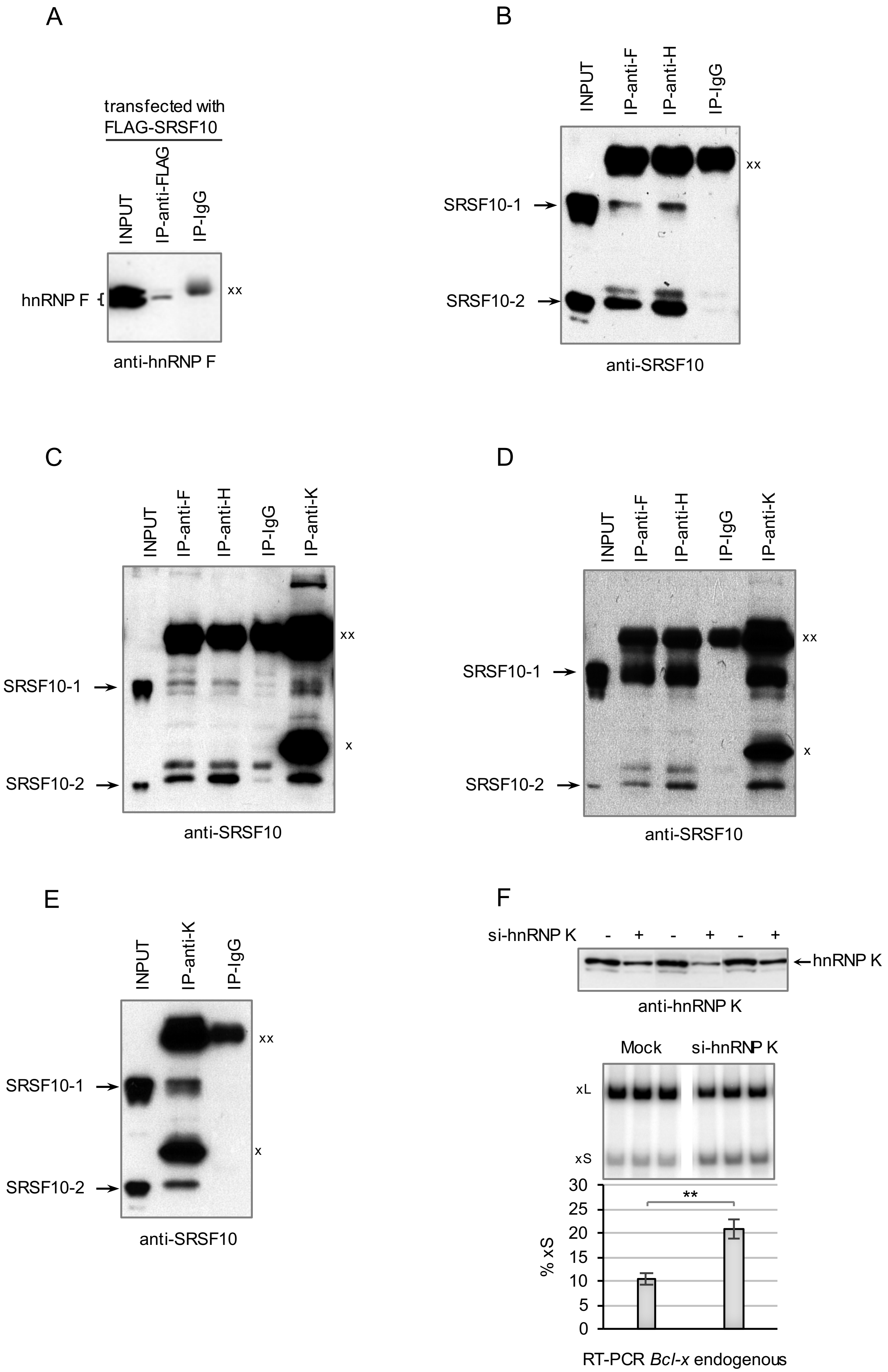

Supplemental Figure 2

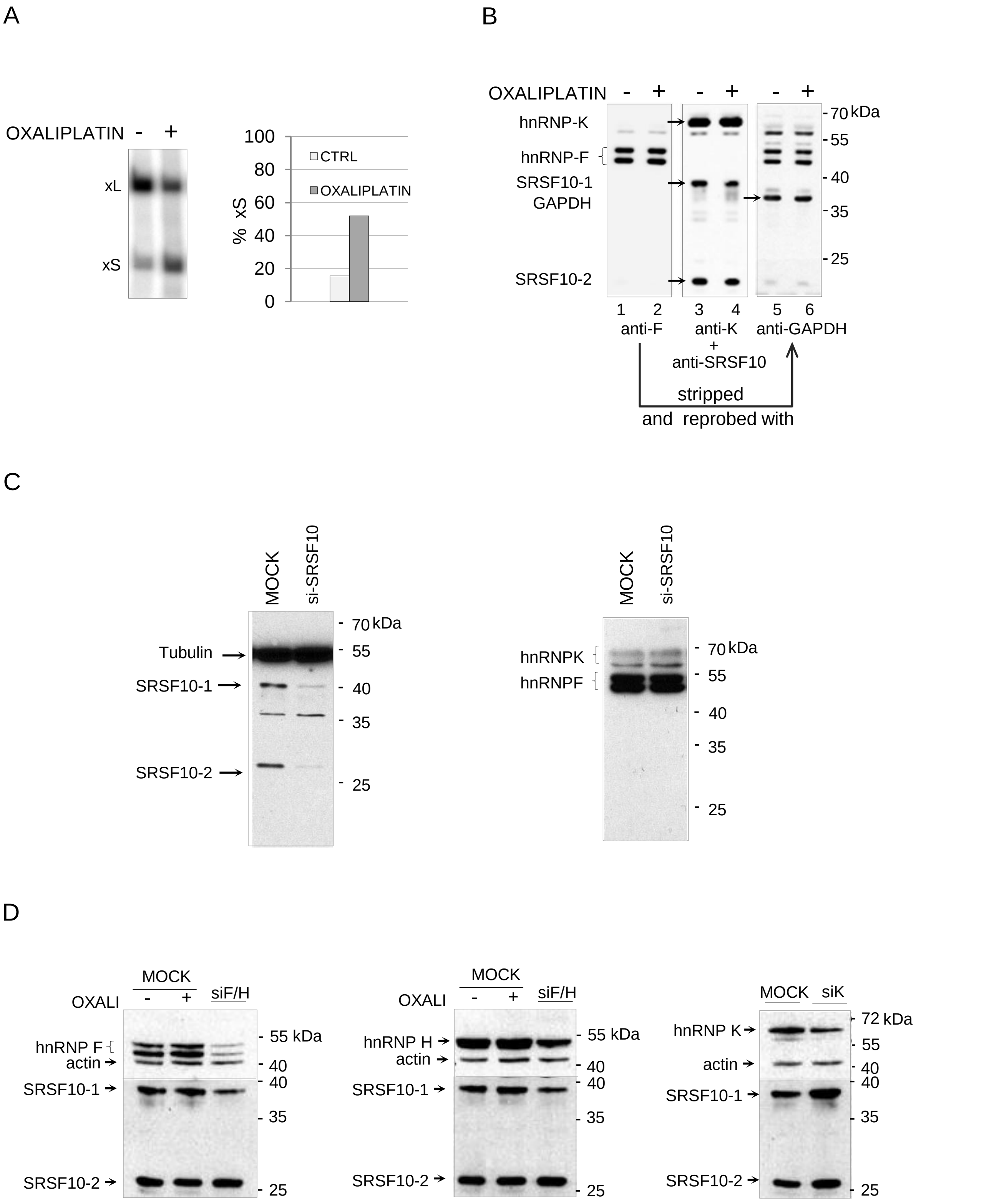

Supplemental Figure 3

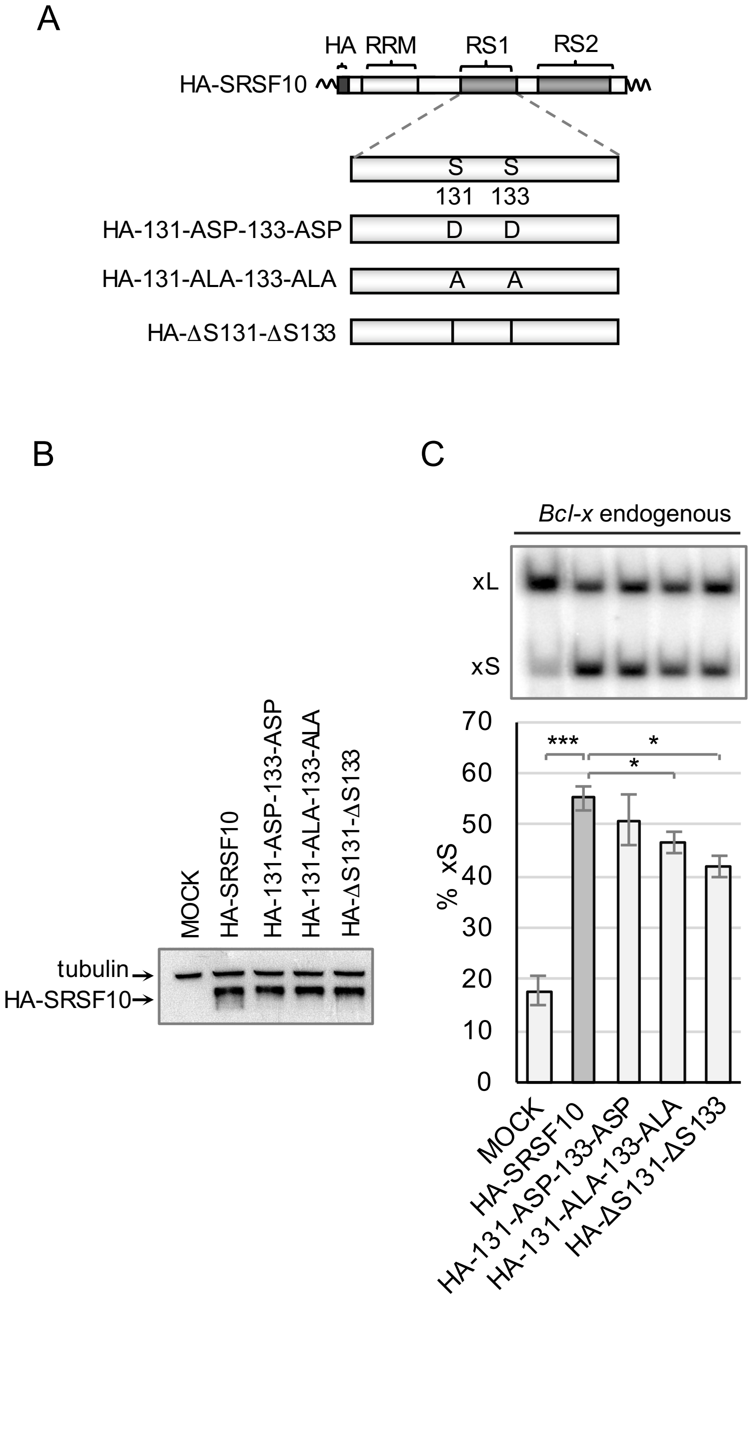

Supplemental Figure 4

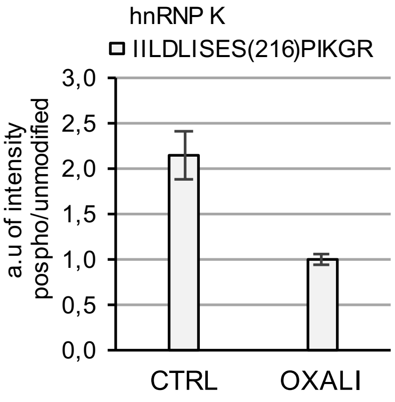

Supplemental Figure 5

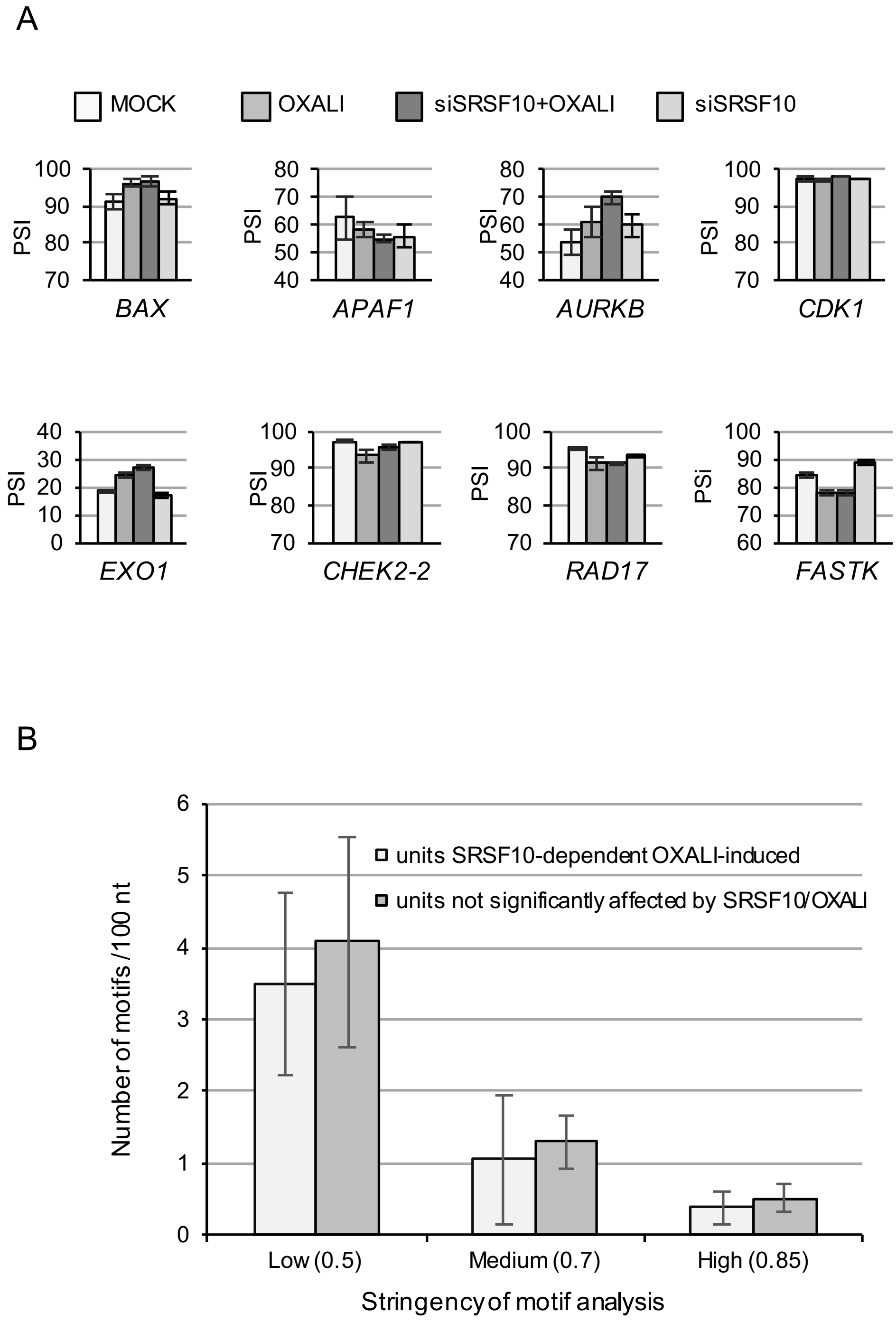

Supplemental Figure 6

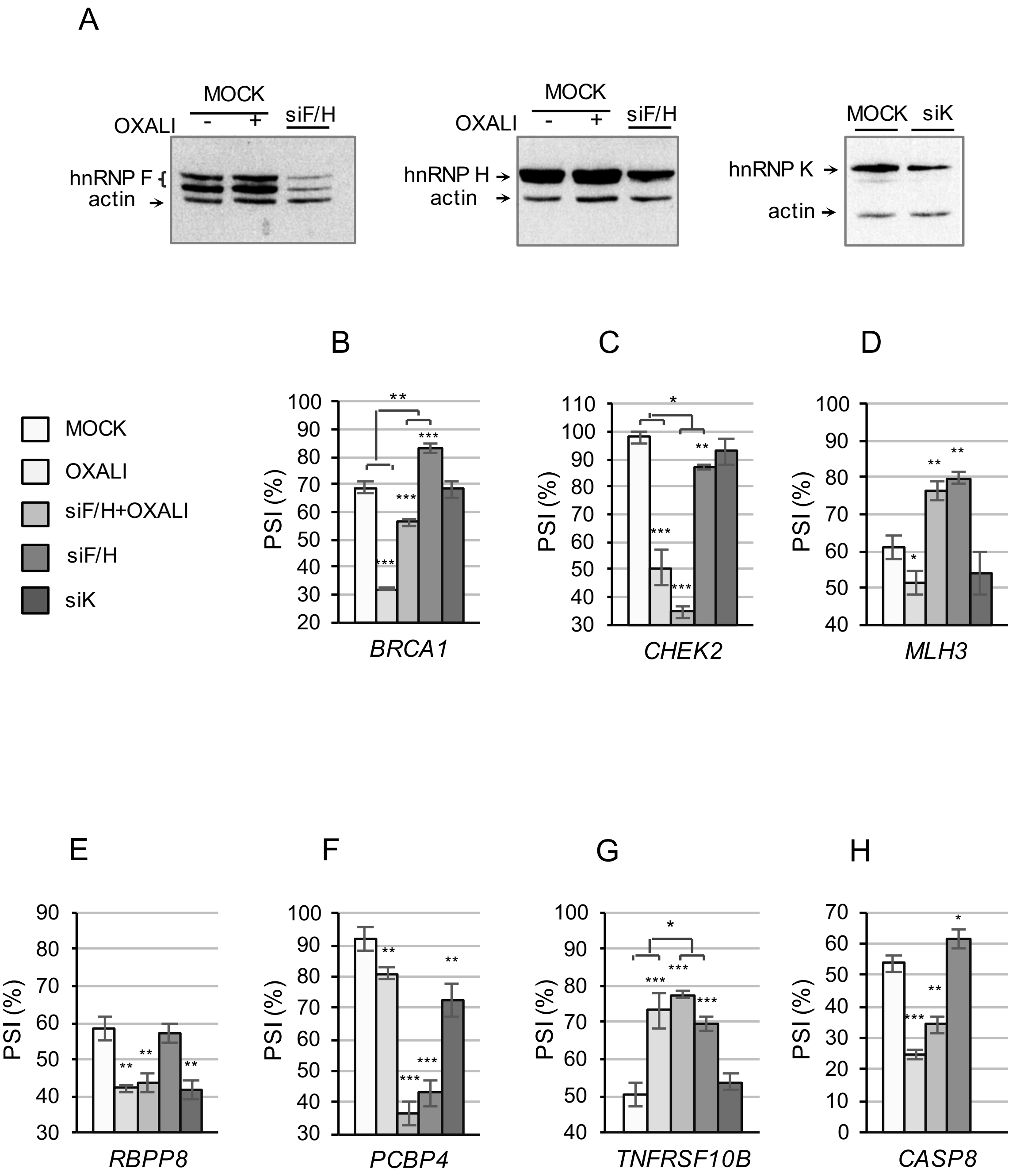

Supplemental Figure 7

Shkreta_Suppl-Fig 8

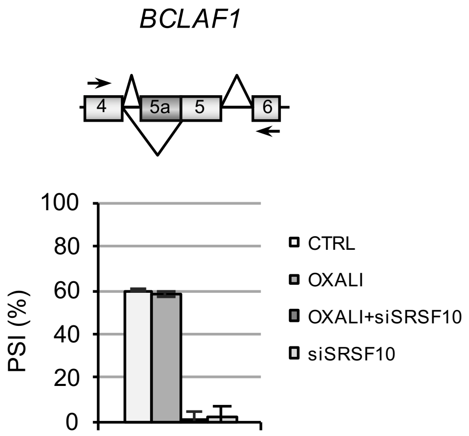

Supplement: supp_guide [file NIHMS868525-supplement-Supp_Material.pdf]
